# Cardioprotective potential of Maharishi Amrit Kalash: a scoping review of evidence from experimental and clinical studies

**DOI:** 10.3389/fphar.2026.1799117

**Published:** 2026-05-14

**Authors:** Radha Singh, Rini Vohra, Richa Shrivastava, Robert H. Schneider

**Affiliations:** 1 Maharishi Ayurveda Products Private Limited, Noida, Uttar Pradesh, India; 2 Maharishi University of Information Technology, Noida, Uttar Pradesh, India; 3 Maharishi Ayurveda Europe, Roermond, Netherlands; 4 Institute for Natural Medicine and Prevention, Maharishi International University, Fairfield, IA, United States

**Keywords:** angina, atherosclerosis, cardiovascular diseases, heart, Maharishi Amrit Kalash, MAK

## Abstract

**Introduction:**

Cardiovascular disease (CVD) remains the leading cause of mortality worldwide. Maharishi Amrit Kalash (MAK) is a standardized Ayurvedic formulation developed within the framework of *Brahma Rasayana* and has been investigated in experimental and clinical studies for its potential cardiovascular benefits. However, the scope and nature of the available evidence supporting its role in cardiovascular disease have not been systematically mapped. This scoping review aims to summarize the existing research on the role of MAK in cardiovascular disease and to identify current evidence gaps, following the PRISMA-ScR guidelines.

**Methods:**

This scoping review was conducted in accordance with the PRISMA-ScR framework. A comprehensive literature search was performed across multiple databases, including PubMed (n = 25), AYUSH Research Portal (n = 7), Google Scholar (n = 208), DHARA (n = 3), EBSCO (n = 3), and the Clinical Trials Registry of India (CTRI; n = 5). Preclinical and clinical studies evaluating MAK in relation to atherosclerosis, angina, ischemic heart disease, plaque formation, and other relevant cardiovascular outcomes were screened using predefined inclusion and exclusion criteria.

**Results:**

Out of 82 identified records, 13 studies met the inclusion criteria. *In vitro* studies consistently demonstrated inhibition of Cu^2+^-induced low-density lipoprotein (LDL) oxidation, suppression of enzymatic and non-enzymatic lipid peroxidation, and modulation of platelet aggregation induced by various agonists. *In vivo* studies reported reduced atheromatous plaque formation, increased myocardial glutathione levels, and attenuation of oxidative stress markers in cardiac tissue. Clinical studies indicated reductions in the frequency and severity of angina, improvements in exercise tolerance, and increases in left ventricular ejection fraction in patients with ischemic heart disease. Across study types, antioxidant and anti-atherogenic mechanisms were the most frequently investigated outcomes.

**Discussion:**

This scoping review provides a structured overview of the available experimental and clinical evidence on MAK in cardiovascular disease, highlighting recurring mechanistic pathways and clinical endpoints. While the findings support the biological plausibility of antioxidant and anti-atherogenic actions, substantial heterogeneity and methodological limitations are evident across the included studies. These findings underscore the need for well-designed randomized controlled trials to establish the clinical efficacy and therapeutic relevance of MAK in the prevention and management of atherosclerotic cardiovascular disease.

## 1 Introduction

Cardiovascular disease (CVD) accounts for approximately 17.9 million deaths globally, with India contributing nearly 20% of this burden and a disproportionate impact on younger populations (Jan et al., 2024; World Health Organisation, 2021). The current CVD burden is characterized by increased risk exposure, earlier disease onset, higher premature mortality, and elevated fatality rates (Floria et al., 2026; Zhu et al., 2025). These trends are largely associated with population growth, urbanization, and lifestyle-related stressors (Teo and Rafiq, 2021). Despite advances in pharmacological management, long-term treatment costs, extended duration of therapy, and potential adverse effects contribute to limited accessibility and poor treatment adherence, particularly in resource-constrained settings (Namas et al., 2025; Centers for Disease Control and Prevention, 2018). These challenges highlight the need for complementary approaches that support long-term cardiovascular risk management. In this context, the adoption of a holistic and pragmatic approach to CVD prevention and management becomes imperative (Ghoshal et al., 2026; Gupta and Mehta, 2026; Alsaidan, 2025; Caturano et al., 2025; Ekor, 2014; Mukherjee, 2012).

Traditional systems of medicine, including Ayurveda, have been explored as adjunctive strategies for chronic disease prevention and management (Niharika et al., 2026; Balkrishna et al., 2026; World Health Organisation, 2022; Chauhan et al., 2015). Within Ayurveda, Rasayana refers to therapeutic approaches aimed at maintaining physiological balance, supporting tissue nourishment, and preserving functional capacity over time (Bhati et al., 2025; Goyal, 2018). Rasayana interventions are traditionally described as influencing the quality of Rasa (circulating bodily fluids) and the subsequent Dhatus (tissues), thereby supporting systemic resilience and overall health (Sharma, 1983). Among Rasayana formulations, Brahma Rasayana is a classical preparation described in the *Charaka Samhita* (Chikitsa Sthana) (Shastri, 1975) and is considered a systemic rejuvenative therapy intended to support vitality, tissue nourishment, and resistance to disease. Its broader physiological relevance provides a conceptual framework for exploring such formulations in chronic, multifactorial conditions, including cardiovascular disease (Government of India, 2003).

Maharishi Amrit Kalash (MAK) is a modern Ayurvedic formulation developed based on the Brahma Rasayana concept. Rasayana therapies, including MAK, are traditionally associated with promoting systemic nourishment, physiological balance, and maintenance of vitality. MAK is generally administered as two separate, complementary preparations, MAK-4 and MAK-5, while MAK-7 represents a modified version (sugar free) of MAK-4 formulated without ghee, cane sugar, and honey and available in tablet form. These are recommended to be administered together (MAK-4 followed by MAK-5).

MAK-4 is formulated as a semi-solid paste. It is prepared in the base of Amla and Harad Pishti (cooked with sugar) and other powdered botanical drugs are added to them and then cooked over a period of 5–6 days. While, MAK-5 is prepared in tablet form. The formulation involves blending the dried powdered forms of 13 botanical drugs followed by its compression into tablets. The complete list of botanical drugs used in both formulations is provided in Table 1.

**TABLE 1:** Botanical drugs used in preparation of MAK-4 and MAK-5 and their reported pharmacological properties.

S. No.	Botanical drug used	Botanical name in registered formulation	Part used	Composition in %	Taxonomic validation and family	Medicinal source (published)	Medicinal use	References
MAK-4								
1	Bilva	*Aegle marmelos*	Bark	0.227	Rutaceae*; Aegle marmelos (L.) Corrêa*	Ayurvedic Pharm. Of India (1999-2011) Global substance Registration system (GSRS) (FDA/NCATS, 2024)	Antidiarrhoeal, digestive, antidiabetic, antioxidant, hepatoprotective	Monika et al. (2023)
2	Sonapatha	*Oroxylum indicum*	Bark	0.227	Bignoniaceae*; Oroxylum indicum (L.) Kurz*	Global substance Registration system (GSRS) (FDA/NCATS, 2024)	Anti-inflammatory, antioxidant, used for respiratory ailments, wound healing	Jagetia (2021)
3	Gambhari	*Gmelina arborea*	Bark	0.227	Lamiaceae*; Gmelina arborea Roxb. ex Sm*	Ayurvedic Pharm. Of India (1999-2011) Global substance Registration system (GSRS) (FDA/NCATS, 2024)	Astringent, digestive, cardiotonic, nervine tonic; used in fever, wounds	Warrier et al. (2021)
4	Padal (Patala)	*Stereospermum suaveolens*	Bark	0.227	Bignoniaceae*; Stereospermum chelonoides (L.f.) DC.*	Ayurvedic Pharm. Of India (1999-2011) Global substance Registration system (GSRS) (FDA/NCATS, 2024)	Analgesic, anti-inflammatory, used in Dashamoola; wound healing, antipyretic	Lade et al. (2022)
5	Arni	*Clerodendrum phlomidis*	Whole plant	0.227	Lamiaceae*; Clerodendrum phlomidis L.f*	Ayurvedic Pharm. Of India (1999-2011) Herbs of commerce (McGuffin et al., 2000) U.S. FDA substance Registration system (2016)	Bitter tonic, anti-inflammatory, used in respiratory disorders, fever, rheumatism	Mohan Maruga Raja et al. (2010)
6	Triparni	*Desmodium gangeticum*	Whole plant	0.227	Fabaceae*; Pleurolobus gangeticus (L.) J.St.-Hil. ex H.Ohashi and K.Ohashi*	Med. Pl. of the world (wyk and wink, 2004) Indian Med. Pl. Database (TDU, 2020)	Tonic, anti-inflammatory, antioxidant, used in Dashamoola, respiratory and digestive ailments	Rastogi et al. (2011)
7	Prishniparni	*Uraria picta*	Whole plant	0.227	Fabaceae*; Uraria picta (Jacq.) Desv. ex DC.*	Indian Med. Pl. Database (TDU, 2020) Global substance Registration system (GSRS) (FDA/NCATS, 2024)	Used in Dashamoola: anti-inflammatory, wound-healing, immunomodulatory; used for cough/fever/diarrhoea	Vats et al. (2024)
8	Kantkari (badi)	*Solanum indicum*	Whole plant	0.227	*Solanacae; Solanum indicum*	Ayurvedic Pharm. Of India (1999-2011)	Expectorant, bronchodilator, used in respiratory conditions; antimicrobial activity reported	Sharma et al. (2017)
9	Kantkari (chhoti)	*Solanum xanthocarpum*	Whole plant	0.227	Solanaceae*; Solanum xanthocarpum L*	Indian Med. Pl. Database (TDU, 2020)	Respiratory disorders, expectorant, anti-asthmatic; used in cough and bronchitis	Iqubal et al. (2022)
10	Gokharu bada	*Pedalium murex*	Fruit	0.227	Pedaliaceae*; Pedalium murex L*	Demand and Supply of Med. Pl. in India (ved et al., 2008) Global substance Registration system (GSRS) (FDA/NCATS, 2024)	Traditionally used for urinary/renal complaints, diuretic and tonic	Ramadevi et al. (2020)
11	Gokharu chhota	*Tribulus terrestris*	Fruit	0.227	Zygophyllaceae*; Tribulus terrestris L*	Ayurvedic Pharm. Of India (1999-2011) Indian Med. Pl. Database (TDU, 2020) Global substance Registration system (GSRS) (FDA/NCATS, 2024)	Diuretic, urinary tract support, aphrodisiac, cardiotonic antioxidant	Ștefănescu et al. (2020)
12	Mudgaparni	*Phaseolus trilobus*	Whole plant	0.227	Fabaceae*; Pueraria montana* var. *lobata (Willd.) Maesen and S.M.Almeida ex Sanjappa and Predeep]*	Indian Med. Pl. Database (TDU, 2020)	Hepatoprotective and antioxidant	Fursule and Patil (2010)
13	Masparni	*Teramnus labialis*	Whole plant	0.227	Fabaceae*; Teramnus labialis (L.f.) Spreng*	Demand and Supply of Med. Pl. in India (ved et al., 2008) Global substance Registration system (GSRS) (FDA/NCATS, 2024)	Rasayana/immunomodulatory, cardioprotective activities reported; antioxidant/anti-inflammatory	Rakshe et al. (2024)
14	Erand	*Ricinus communis*	Root	0.227	Euphorbiaceae*; Ricinus communis L*	Ayurvedic Pharm. Of India (1999-2011) U.S. FDA substance Registration system (2016)	Purgative (castor oil), laxative, used topically for inflammation/pain (oil), emmenagogue historically	Jena and Gupta (2012)
15	Bala	*Sida cordifolia*	Whole plant	0.227	Malvaceae*; Sida cordifolia L*	Demand and Supply of Med. Pl. in India (ved et al., 2008) Global substance Registration system (GSRS) (FDA/NCATS, 2024)	Nervine tonic, analgesic, anti-inflammatory, used for debility and respiratory complaints	Hasan et al. (2025)
16	Kans	*Saccharum spontaneum*	Root	0.227	Poaceae*]; Saccharum spontaneum L*	Ayurvedic Pharm. Of India (1999-2011) Global substance Registration system (GSRS) (FDA/NCATS, 2024)	Used as astringent, diuretic; traditional uses for burning sensations, piles, gynecological and urinary disorders	Hassan et al. (2024)
17	Kush	*Eragrostis cynosuroides*	Root	0.227	Poaceae*; Desmostachya bipinnata (L.) Stapf*	Ayurvedic Pharm. Of India (1999-2011)	Ritual grass with traditional uses; some reports of antimicrobial/medicinal uses in folk medicine	Joshi and Kumar (2024)
18	Satavar	*Asparagus racemosus*	Root	0.227	Asparagaceae*; Asparagus racemosus Willd*	Ayurvedic Pharm. Of India (1999-2011) Global substance Registration system (GSRS) (FDA/NCATS, 2024)	Rejuvenative (rasayana) — galactagogue, female tonic, adaptogen, antioxidant	Alok et al. (2013)
19	Punarnava	*Boerhaavia diffusa*	Whole plant	0.227	Nyctaginaceae*; Boerhavia diffusa L*	Ayurvedic Pharm. Of India (1999-2011) Global substance Registration system (GSRS) (FDA/NCATS, 2024)	Diuretic, hepatoprotective, anti-inflammatory; used for oedema and urinary disorders	Das et al. (2023)
20	Jivanti	*Leptadenia reticulata*	Whole plant	0.227	Apocynaceae*; Leptadenia reticulata (Retz.) Wight and Arn*	Indian Med. Pl. Database (TDU, 2020) Global substance Registration system (GSRS) (FDA/NCATS, 2024)	Rasayana, cardiotonic, used for respiratory disorders, wound healing, immunomodulator	Mohanty et al. (2017)
21	Ikshu/Chini	*Saccharum officinarum*	Root	0.227	Poaceae*; Saccharum officinarum L*	Ayurvedic Pharm. Of India (1999-2011) Global substance Registration system (GSRS) (FDA/NCATS, 2024)	Sweet, nutritive, coolant; used as base (sugar) and in formulations; demulcent and tonic	Ashwini et al. (2022)
22	Manichhidra	*Polygonatum verticillatum*	Root	0.227	Asparagaceae*; Polygonatum verticillatum (L.) All*	Global substance Registration system (GSRS) (FDA/NCATS, 2024)	Rasayana/nutritive, used for prevention of CVDs, for weakness and aphrodiasic	Lin et al. (2024)
23	Kshir vidari	*Ipomoea digitata*	Tuberous root	0.227	Convolvulaceae*; Ipomoea digitata L*	Ayurvedic Pharm. Of India (1999-2011) Global substance Registration system (GSRS) (FDA/NCATS, 2024)	Rejuvenative, nutritive, tonic; cardioprotective	Srivastava (2020)
24	Vidari	*Pueraria tuberosa*	Tuberous root	0.227	Fabaceae*; Pueraria tuberosa (Roxb. ex Willd.) DC.*	Ayurvedic Pharm. Of India (1999-2011) Global substance Registration system (GSRS) (FDA/NCATS, 2024)	Rasayana, galactagogue, aphrodisiac, antioxidant, hepatoprotective	Bharti et al. (2021)
25	Amla pishti (with 40% ghee)	*Phyllanthus emblica*	Fruit	13.000	Phyllanthaceae*; Phyllanthus emblica L*	Ayurvedic Pharm. Of India (1999-2011) Global substance Registration system (GSRS) (FDA/NCATS, 2024)	Rejuvenative, antioxidant, rich in vitamin C; support vascular health, immunity and as rasayana	Almatroodi et al. (2020)
26	Harad pishti (with 40% ghee)	*Terminalia chebula*	Fruit	6.500	Combretaceae*; Terminalia chebula Retz*	Ayurvedic Pharm. Of India (1999-2011) Global substance Registration system (GSRS) (FDA/NCATS, 2024)	Digestive, mild laxative, rejuvenative in formulations; antioxidant	Harminder et al. (2011)
27	Dalchini	*Cinnamomum zeylanicum*	Bark	0.398	Lauraceae*; Cinnamomum verum J.Presl*	Ayurvedic Pharm. Of India (1999-2011)	Carminative, digestive, anti-microbial, metabolic (insulin-sensitivity) effects reported	Singh et al. (2021)
28	Elaichi chhoti	*Elettaria cardamomum*	Fruit	0.398	*[*Zingiberaceae*; Elettaria cardamomum (L.) Maton*	Indian Med. Pl. Database (TDU, 2020) Global substance Registration system (GSRS) (FDA/NCATS, 2024)	Carminative, digestive, aromatic, used for nausea and digestive complaints	Ashokkumar et al. (2020)
29	Nagarmotha	*Cyperus rotundus*	Tuberous root	0.398	Cyperaceae*; Cyperus rotundus L*	Ayurvedic Pharm. Of India (1999-2011) Global substance Registration system (GSRS) (FDA/NCATS, 2024)	Carminative, digestive, anti-inflammatory; used in dysmenorrhea and digestive disorders	Pirzada et al. (2015)
30	Haldi	*Curcuma longa*	Rhizome	0.398	Zingiberaceae*; Curcuma longa L*	Ayurvedic Pharm. Of India (1999-2011) Pl. Méd. de la Pharmacop.: Liste A (2000)	Anti-inflammatory, antioxidant, hepatoprotective — used broadly as Rasayana/antioxidant	Iweala et al. (2023)
31	Pippali chhoti	*Piper longum*	Fruit	0.398	Piperaceae*; Piper longum L*	Ayurvedic Pharm. Of India (1999-2011) Global substance Registration system (GSRS) (FDA/NCATS, 2024)	Carminative, bronchodilator, digestive stimulant; used in respiratory and digestive preparations	Biswas et al. (2022)
32	Chandan safed	*Santalum album*	Heartwood	0.398	Santalaceae*; Santalum album L*	Ayurvedic Pharm. Of India (1999-2011) Global substance Registration system (GSRS) (FDA/NCATS, 2024)	Cooling, astringent, antioxidant, anticancer, anti-inflammatory, antiviral, antibacterial, antifungal, hepatoprotective and cardio-protective properties	Choudhary and Chaudhary (2021)
33	Brahmi	*Centella asiatica*	Whole plant	0.398	Apiaceae*; Centella asiatica (L.) Urb*	Ayurvedic Pharm. Of India (1999-2011) Global substance Registration system (GSRS) (FDA/NCATS, 2024)	Cognitive tonic, nervine, adaptogen, antioxidant	Boughaleb et al. (2025)
34	Motha desi	*Cyperus scariosus*	Root	0.398	Cyperaceae*; Cyperus scariosus R.Br*	Indian Med. Pl. Database (TDU, 2020) Global substance Registration system (GSRS) (FDA/NCATS, 2024)	Carminative, digestive, used in traditional tonics	Choudhary and Chaudhary (2021)
35	Nagkesar	*Mesua ferrea*	Flower	0.398	Calophyllaceae*; Mesua ferrea L*	Ayurvedic Pharm. Of India (1999-2011) Global substance Registration system (GSRS) (FDA/NCATS, 2024)	Astringent, haemostatic, used in piles, bleeding disorders and wound healing, digestive	Kuerban et al. (2024)
36	Shankhapushpi	*Convolvulus prostratus*	Whole plant	0.398	Convolvulaceae*; Convolvulus prostratus Forssk*	Ayurvedic Pharm. Of India (1999-2011) Global substance Registration system (GSRS) (FDA/NCATS, 2024)	Nervine tonic, memory enhancer, mild sedative/brain tonic	Balkrishna et al. (2020)
37	Mulethi	*Glycyrrhiza glabra*	Root	0.398	Fabaceae*; Glycyrrhiza glabra L*	Ayurvedic Pharm. Of India (1999-2011) Global substance Registration system (GSRS) (FDA/NCATS, 2024)	Expectorant, demulcent, anti-inflammatory, gastric protective	Wahab et al. (2021)
38	Amogha	*Embelia ribes*	Fruit	0.398	Primulaceae*; Embelia ribes Burm.f*	Indian Med. Pl. Database (TDU, 2020)Global substance Registration system (GSRS) (FDA/NCATS, 2024)	Anthelmintic, digestive, used in intestinal worms, digestive complaints	Sharma et al. (2022)
MAK-5								
1	Ashwagandha	*Withania somnifera*	Root	20.20	Solanaceae*; Withania somnifera (L.) Dunal*	Indian Med. Pl. Database (TDU, 2020) Global substance Registration system (GSRS) (FDA/NCATS, 2024)	Adaptogenic, reduces stress, improves strength and immunity	Mikulska et al. (2023)
2	Mulethi	*Glycyrrhiza glabra*	Root	20.20	Fabaceae*; Glycyrrhiza glabra L*	Ayurvedic Pharm. Of India (1999-2011) U.S. FDA substance Registration system (2016)	Anti-inflammatory, throat-soothing, respiratory support	Pastorino et al. (2018)
3	Kshirvidari	*Ipomoea digitata*	Tuberous root	20.20	Convolvulaceae*; Ipomoea digitata L*	Indian Med. Pl. Dictionary (Khare, 2007) Global substance Registration system (GSRS) (FDA/NCATS, 2024)	Rasayana, strength-promoting, hormone-supportive	Rauniyar and Srivastava (2020)
4	Musali Safed	*Chlorophytum borivilianum*	Tuberous root	20.20	Asparagaceae*; Chlorophytum borivilianum Santapau and R.R.Fern*	Demand and Supply of Med. Pl. in India (ved et al., 2008) Global substance Registration system (GSRS) (FDA/NCATS, 2024)	Aphrodisiac, immunomodulatory, vitality enhancer	Pawar et al. (2023)
5	Vidhara	*Argyreia speciosa*	Root	1.01	Convolvulaceae*; Argyreia nervosa (Burm.f.) Bojer*	Ayurvedic Pharm. Of India (1999-2011)	Anti-inflammatory, nervine tonic, reproductive health	Galani et al. (2010)
6	Amla	*Emblica officinalis*	Fruit rind	2.02	Phyllanthaceae*; Phyllanthus emblica L*	Ayurvedic Pharm. Of India (1999-2011) Global substance Registration system (GSRS) (FDA/NCATS, 2024)	Powerful antioxidant, immunity booster, digestive support	Almatroodi et al. (2020)
7	Giloy	*Tinospora cordifolia*	Stem	2.02	Menispermaceae*; Tinospora cordifolia (Willd.) Hook.f. and Thomson*	Demand and Supply of Med. Pl. in India (ved et al., 2008) Global substance Registration system (GSRS) (FDA/NCATS, 2024)	Immunomodulatory, antipyretic, anti-inflammatory	Siddiqui et al. (2025)
8	Shatavari	*Asparagus racemosus*	Root	2.02	Asparagaceae*; Asparagus racemosus Willd*	Ayurvedic Pharm. Of India (1999-2011) Global substance Registration system (GSRS) (FDA/NCATS, 2024)	Female reproductive tonic, galactagogue, adaptogen	Alok et al. (2013)
9	Jalanirgundi	*Vitex trifolia*	Leaf	2.02	*.* Lamiaceae*; Vitex trifolia L*	Indian Med. Pl. Database (TDU, 2020) Global substance Registration system (GSRS) (FDA/NCATS, 2024)	Anti-inflammatory, cardioprotective	Perveen et al. (2023)
10	Shankhpushpi	*Convolvulus pluricaulis*	Whole plant	2.02	Convolvulaceae*; Convolvulus prostratus Forssk*	Ayurvedic Pharm. Of India (1999-2011)	Memory enhancer, anxiolytic, cognitive booster	Sharma et al. (2022)
11	Kali Musli	*Curculigo orchioides*	Tuberous root	2.02	Hypoxidaceae*; Curculigo orchioides Gaertn*	Ayurvedic Pharm. Of India (1999-2011) Global substance Registration system (GSRS) (FDA/NCATS, 2024)	Aphrodisiac, antioxidant, anti-aging	Wang et al. (2021)
12	Karil	*Capparis decidua*	Bark	2.02	Capparaceae*; Capparis decidua (Forssk.) Edgew*	Indian Med. Pl. Database (TDU, 2020) Global substance Registration system (GSRS) (FDA/NCATS, 2024)	Aantioxidant, anti-inflammatory	Nazar et al. (2020)
13	Babool	*Acacia nilotica*	Exudate	4.04	Fabaceae*; Vachellia nilotica (L.) P.J.H.Hurter and Mabb*	World checklist of vascular plants	Antimicrobial, astringent, anti-inflammatory	Hafez et al. (2024)

These Rasayana formulations are described as supporting the functional balance of the seven *Dhatus* (fundamental body tissues in Ayurveda)- *Rasa* (nutritive fluid/plasma responsible for nourishment), *Rakta* (blood tissue involved in oxygenation and vitality), *Mamsa* (muscle tissue responsible for structure and movement), *Meda* (adipose or fat tissue involved in lubrication and energy storage), *Asthi* (bone tissue providing structural support), *Majja* (bone marrow and nervous tissue associated with immunity and neural function), and *Shukra* (reproductive tissue responsible for fertility and vitality)-which collectively represent the major tissue systems of the body (Glaser, 1988; Sharma et al., 1996). Key botanical drugs include *Emblica officinalis*, *Withania somnifera*, *Centella asiatica*, and *Glycyrrhiza glabra*, and phytochemical analyses have reported bioactive metabolites such as polyphenols, carotenoids, and bioflavonoids (Upadhyay and Dixit, 2015; Kamath and Shah, 2014, 2015).

**FIGURE 1:**
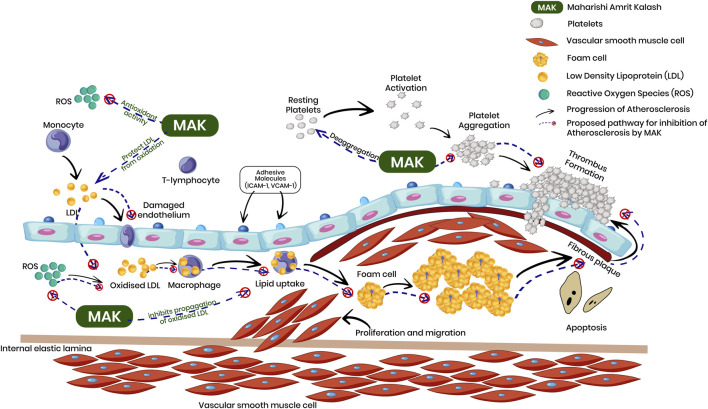
Breaking Down Atherosclerosis with MAK. Schematic representation of the role of oxidative stress in atherosclerosis and the biological pathways investigated for Maharishi Amrit Kalash (MAK).

Experimental studies have primarily explored MAK in relation to antioxidant and immunomodulatory activities, providing a potential mechanistic basis for investigating its relevance in chronic conditions, including cardiovascular disease. Several studies report free-radical scavenging activity and modulation of endogenous antioxidant systems, suggesting possible interactions with cardiovascular mechanisms such as lipid oxidation and endothelial dysfunction (Wang et al., 2026; Zanella et al., 2015; Dwivedi et al., 2005; Scartezzini and Speroni, 2000; Vohra et al., 1999; Bondy et al., 1994). Oxidative stress plays a central role in cardiovascular disease, where excessive reactive oxygen species promote lipid peroxidation and formation of oxidized LDL (Figure 1). Oxidized LDL contributes to endothelial inflammation, monocyte recruitment, macrophage differentiation, and foam-cell formation—key steps in atherosclerotic plaque development (Jiao et al., 2025; Jiang et al., 2022). Platelet aggregation and endothelial activation further accelerate atherosclerosis and related cardiovascular events (Vilahur and Fuster, 2025; Batty et al., 2022). Within this context, MAK has been examined in experimental models and biochemical assays assessing antioxidant capacity and surrogate markers of oxidative stress (Zanella et al., 2015; Vohra et al., 2001; Sharma et al., 1994), though the extent to which these findings translate into clinically meaningful cardiovascular outcomes remains limited.

In addition to cardiovascular-related mechanisms, MAK has been investigated in studies addressing immunomodulatory effects, chemoprotective activity, vitality, and broader aspects of physiological homeostasis (Wankhade and Khalekar, 2012; Saxena et al., 2008; Penza et al., 2007; Inaba et al., 1995; Inaba et al., 1996; Inaba et al., 1997; Gelderloos et al., 1990; Samaiya et al., 1999; Prasad et al., 1993; Patel et al., 1992; Engineer et al., 1992; Sharma et al., 1990; Dwivedi et al., 1988). These investigations encompass diverse experimental models and clinical contexts, reflecting the wide range of biological effects that have been explored for this formulation. Nevertheless, the studies vary considerably in experimental design, model systems, outcome measures, and reporting standards, which complicates interpretation of the overall evidence base.

Given the heterogeneous and dispersed nature of the available literature, a structured synthesis is necessary to map the scope and characteristics of existing research. A scoping review is particularly suited to this purpose, as it allows systematic identification and categorization of studies, highlights gaps in knowledge, and clarifies areas where further investigation is warranted (Munn et al., 2018).

Although previous scoping reviews have examined MAK in other contexts, including oncology-related applications (Vohra et al., 2024; Koch et al., 2024) and immunomodulatory actions (Singh et al., 2025), no review to date has specifically evaluated the evidence related to cardiovascular disease. The present study therefore aims to systematically map preclinical and clinical investigations of MAK in cardiovascular contexts and to critically examine the characteristics of the available studies, including their experimental design, models used, and outcome measures. By doing so, this review seeks to identify gaps in the current evidence base and inform priorities for future research.

## 2 Methods

### 2.1 Scoping review framework

This study was conducted as a scoping review to map the available evidence on the cardioprotective effects of MAK. Scoping reviews are particularly useful for exploring emerging or heterogeneous fields of research, identifying knowledge gaps, and summarizing the breadth of available literature before conducting systematic reviews or meta-analyses (Peters et al., 2020).

The review followed the methodological framework proposed by Arksey and O’Malley (2005), which outlines five key stages: (1) identifying the research question, (2) identifying relevant studies, (3) study selection, (4) charting the data, and (5) collating, summarizing, and reporting the results. Methodological refinements suggested by Danielle Levac and colleagues (Levac et al., 2010) were also considered to enhance transparency and rigor.

Reporting of the review was guided by the PRISMA Extension for Scoping Reviews (PRISMA-ScR) checklist to ensure systematic reporting of the search process, eligibility criteria, and evidence synthesis (Mak and Thomas, 2022; Tricco et al., 2018) (Supplementary Table S1).

### 2.2 Eligibility criteria

The eligibility criteria were predefined in the study protocol. Studies were included if they evaluated the cardioprotective properties of MAK, including effects on cardiovascular conditions such as atherosclerosis, LDL oxidation, platelet aggregation, angina, myocardial infarction, ischemic heart disease, or related cardiovascular outcomes.

Eligible study designs included *in vitro* experiments*, in vivo* studies, and clinical investigations published in peer-reviewed journals or conference proceedings. There were no restrictions on the year of publication. Only studies published in English and specifically evaluating MAK-4 and/or MAK-5 (Supplementary Table S2) were included.

The following were excluded: case reports, editorials, letters, testimonials, unpublished reports, non-English publications, handwritten reports, and abstracts with insufficient or inconclusive data. These criteria were applied consistently to ensure that the included studies were directly relevant to the cardiovascular effects of MAK.

### 2.3 Information sources

A comprehensive literature search was conducted across multiple electronic databases, including PubMed, Google Scholar, Scopus, ScienceDirect, EBSCO, AYUSH Research Portal, DHARA Ayurvedic Research Database, ClinicalTrials.gov, and the Clinical Trials Registry of India (CTRI).

The search strategy included the following keywords and combinations: *Maharishi Amrit Kalash, Amrit Kalash, MAK-4, MAK-5, MAK-7, M4, M5, M7,* and *Amrit Nectar and Amrit Kalash Ambrosia.*

### 2.4 Search strategy

Studies published between 1988 and 2025 were considered for inclusion. The search strategy was designed to capture both indexed and non-indexed literature relevant to MAK. Reference lists of all included articles were manually screened to identify additional studies.

For full texts that were not publicly accessible, institutional libraries, resources from Maharishi International University, and related websites were consulted. No grey literature was included in this scoping review. When necessary, corresponding authors were contacted to obtain additional data or clarifications. Information on ongoing or unpublished clinical trials was obtained by contacting principal investigators listed in trial registries.

### 2.5 Selection of evidence

Two reviewers (RS1 and RV) independently screened all retrieved records, including *in vitro, in vivo*, and clinical studies. Titles and abstracts were first screened for relevance, followed by full-text evaluation.

Each study was categorized in an Excel-based screening table as “Include,” “Exclude,” or “Uncertain.” Any discrepancies or uncertain cases were resolved through discussion with additional reviewers (RS2 and RS3) until consensus was achieved. Only full-text articles and conference abstracts with sufficient methodological details were included in the final analysis.

### 2.6 Data charting

Data extraction was performed using a standardized data charting form. RS2 and RS3 verified the extracted information to ensure completeness and accuracy.

Key information extracted from each study included study design, population or experimental model, intervention, comparator, and outcomes, following the PICO framework. Data were organized into structured evidence tables using Microsoft Excel. Digital collaboration tools such as Google Drive and Google Docs were used for document management and team coordination.

### 2.7 Synthesis of results

All included studies were summarized in a PICO evidence table (Table 2), highlighting study characteristics and key outcomes. Studies were categorized according to design (*in vitro, in vivo*, or clinical) and analyzed based on the cardiovascular mechanisms or endpoints evaluated.

**TABLE 2:** Characteristics of preclinical and clinical studies evaluating effectiveness of Maharishi Amrit Kalash (MAK) for its cardioprotective properties (PICO table).

Design	Patient population	Dosage	Intervention	Outcomes	Conclusion	Category	Type of MAK used	Cardiac protection type	Abstract/Full paper	References
Preclinical studies										
*In vivo*	Adult male, Sprague–Dawley rats (250–350 g)	0.1% solution of MAK-4	The role of MAK-4 on functional and biochemical integrity of isolated rat heart during oxidative stress induced by H_2_O_2_ was determined in a modified Langendorff model In another experiment cardiac function was assessed by measuring developed contractile tension	Administered with H_2_O_2_, MAK-4 reduced glutathione release, though levels were higher than control or MAK-4 alone. MAK-4 exposure doubled LDH levels, mitigated by adding MAK-4 to H_2_O_2_-perfused hearts. Introducing MAK-4 (0.1%) rapidly decreased pro-oxidant concentration by almost 50%. Importantly, MAK-4 alone did not induce contractile failure during testing	MAK-4 demonstrated a protective effect against oxidative stress, as indicated by reduced LDH and glutathione release in H2O2-containing perfusate. Additionally, its presence improved tension development in H_2_O_2_-treated hearts, suggesting a potential cardioprotective role under varied experimental conditions	Oxidative stress in hearts	MAK-4	Strengthening of heart muscle and decrease in contractile tension	Full paper	Cullen et al. (1997)
*In vitro*	LDL isolated from blood of hyperlipidaemic patients	10 g MAK-4 and 500 mg MAK-5	Investigation of role of MAK-4 and 5 in inhibition of endothelial cell and soyabean lipoxygenase induced LDL oxidation	Alcoholic extracts from MAK-4 and MAK-5 effectively inhibited LDL oxidation induced by endothelial cells and soybean lipoxygenase, with IC50 values of 150 ± 10 and 488.3 ± 41.9 nmol/μg, respectively. Both extracts showed free radical scavenging activity, with values of 16.35 ± 4.27 and 3.64 ± 1.24 nmol/μg for MAK-4 and MAK-5, respectively	MAK-4 and MAK-5 protects LDL from oxidation that could be a benefit in prevention and treatment of atherosclerosis	Atherosclerosis Hyperlipidaemia	MAK-4 and MAK-5	Resistance of human LDL oxidation	Full Paper	Sharma et al. (1994)
*In vitro* *+In vivo*	Blood drawn from hyperlipidaemic patients	10 g MAK-4 and 500 mg MAK-5	Evaluation of *in vivo* antioxidant activity of MAK-4 in hyperlipidaemic patients Plasma lipid profile, plasma peroxides and the protective effect of MAK-4 (study period 6 months)	Administration of MAK-4 for 1.5, 3, 4.5, and 6 months significantly prolonged the lag in LDL oxidation by 6, 6, 10, and 24 h, respectively (baseline 0 h). There was no significant change in plasma peroxide levels. Patients with a high lipid profile exhibited increased resistance to LDL oxidation	MAK-4 demonstrates potential for both the prevention and treatment of atherosclerosis and vascular damage by effectively increasing resistance to LDL oxidation	Atherogenesis	​	Resistance to LD: oxidation Hyperlipidaemia Vascular damage	Abstract	Sundaram et al. (1995)
*In vitro*	Platelet rich plasma isolated from healthy volunteers fasted overnight	600 mg of MAK-4 and 600 mg MAK-5 separately in 2.4 mL saline	Platelet aggregation in whole blood was done by dual channel aggregometer Aggregation test was performed by impedance method	Platelet aggregation significantly reduced with arachidonic acid (53.9 ± 2.0, p < 0.001) and collagen (18.6 ± 2.1, p < 0.001), and was completely inhibited with epinephrine (0%). Full reversal of ADP-induced aggregation occurred, and second-phase aggregation caused by epinephrine was prevented. In whole blood (0%, p < 0.001 in collagen; 22.4% ± 7.2%, p < 0.05 in ADP), platelet aggregation decreased with these chemicals	MAK has the capacity to inhibit platelet aggregation triggered by different groups of aggregator chemicals. This capability holds potential benefits in conditions like hyperlipidaemia, hypertension, diabetes mellitus, cigarette smoking, and stress, where platelets tend to be more prone to aggregation	Cardiovascular disease Hyperlipidaemia	MAK-4 and MAK-5	Platelet aggregation	Full paper	Sharma et al. (1989)
*In vitro*	LDL isolated from human blood samples	50 mg MAK-4 and MAK-5 separately in 25 mL Ham’s F-10 media	Evaluating the impact of MAK-4 and MAK-5 (M-4 and M-5) on LDL oxidation, and comparing their effectiveness to ascorbic acid, α-tocopherol, and probucol	The MAHM exhibited greater antioxidant potency in preventing LDL oxidation compared to ascorbic acid, α-tocopherol, or probucol. Additionally, MAHMs effectively inhibited both the initiation and propagation phases of cupric ion-catalysed LDL oxidation	The antioxidant strength of both MAK-4 and MAK-5 extracts surpassed that of ascorbic acid, α-tocopherol, and probucol, suggesting potential synergistic effects. Alcoholic extracts, rich in lipophilic antioxidants, exhibited superior efficacy compared to their aqueous counterparts	Atherosclerosis	MAK-4 and MAK-5	Prevention of free radical induced injury that led to atherosclerosis	Full paper	Sharma et al. (1992)
*In vitro*	Microvascular endothelial cells (EC) isolated from the choriocapillaris of human eyes	100 mg alcoholic extract of MAK-4 and MAK-5 respectively	Inhibition of EC- and SLP-induced LDL oxidation and SLP-induced Linoleic acid oxidation by MAK4 and 5	Both aqueous and alcoholic extracts of MAK-4 effectively inhibited SLP-induced LDL oxidation in a concentration-dependent manner, with IC50 values of 840.6 ± 196.6 nmol/μg and 246.4 ± 32.5 nmol/μg, respectively. The aqueous extract of MAK-S also showed inhibition of SLP-induced LDL oxidation (IC50: 503.0 ± 139.4 nmol/μg), while the alcoholic extract did not	Both the aqueous and alcoholic extracts of MAK-4 and MAK-5 inhibited EC-induced LDL oxidation	Atherosclerosis	MAK-4 and MAK-5	Prevention of vascular damage	Full paper	Hanna et al. (1996)
*In vivo*	WHHL rabbit	2% MAK-4 (w/w)	Investigation of antiatherogenic and antioxidant activities of MAK. Inhibition of LDL oxidation and plasma lipid peroxidation	LDL treated with MAK-4 displayed a 6-h lag in oxidation, and LDL from MAK-fed rabbits exhibited increased resistance to oxidation. Plasma peroxide levels were comparable between the control and MAK groups. MAK-fed rabbits showed a significant reduction in atheroma size compared to controls (20.7% ± 3.7% vs. 34.5% ± 5.2%)	MAK-4 increases the resistance of LDL to oxidation and prevents atheroma development. The results suggest a role for MAK in increasing resistance to LDL oxidation and preventing atherosclerosis	Atheroma reduction	MAK-4	Antiatherogenic	Abstract	Hanna et al. (1995)
*In vivo*	Watanabe heritable hyperlipidemic (WHHL) rabbits controls (n = 5) And a group fed 6% (w/w) MAK-4 (n = 6)	4% MAK-4 (v/w)	Inhibition of Cu^+^ induced LDL oxidation, and enzymatic- and nonenzymatic induced microsomal lipid peroxidation with MAK-4	In the MAK-4 group, the aortic arch plaque area significantly decreased (22.5% ± 4.2%, mean ± SE) compared to controls (47.6% ± 6.8%, mean ± SE; p < 0.01). MAK-4 also reduced lipid peroxide, increased glutathione peroxidase, and enhanced LDL resistance to oxidation (p < 0.05). Additionally, it lowered the average thoracic aorta atheroma by 49% (p < 0.05) and aortic arch atheroma by 53% (p < 0.01)	MAK-4 reduces atheroma formation, reduces LDL oxidation through its antioxidant activity	Antioxidant and antiatherogenic effect	MAK-4	Antiatherogenic Prevent formation of foam cells by inhibiting LDL oxidation	Full paper	Lee et al. (1996)
*In vivo*	Twenty-four New Zealand white rabbits a control group with normal chow supplement and a test group of 12, fed chow with 4% MAK-4	4% MAK-4	An examination of blood drawn from rabbits included the assessment of superoxide dismutase (SOD), cholesterol, triglycerides, HDL, CK, LD, CK isoenzymes, and LD isoenzymes. Serum lipid peroxide was measured using thiobarbituric acid-reactive substances (TBARS), and TBARS and electrophoretic mobilities were assayed in LDL and VLDL fractions	Throughout the conditioning and regression phases, total CK levels in both groups remained stable, only to surge by 3.3-fold after 12 weeks (p < 0.05). In the control group, CK-BB and CK-MM increased by 4.8-fold (p < 0.05) and 1.4-fold (p < 0.17), respectively. Notably, the test group exhibited significantly lower TBARS concentrations in LDL and VLDL by 10-fold (p < 0.001) and 8-fold (p < 0.001), respectively, compared to the control group. Additionally, the electrophoretic mobilities of LDL and VLDL in the control group were 73% (p < 0.001) and 68% (p < 0.001) higher, respectively, than those in the test group	Incorporating MAK-4 into the diet demonstrates a preventive effect against lipid peroxidation and safeguards cell membranes from free radical-induced damage including heart. These findings suggest a promising application for MAK-4 in conferring an anti-atherosclerotic effect	Hyperlipidaemia and antioxidant property	MAK-4	Antiatherogenic Free radical scavenging	Full paper	Lee et al. (1994)
Clinical studies										
Clinical (Open-label)	30 patients with angina pectoris (32–59 years) 20 males and 10 females	10 g MAK-4 and 500 mg MAK-5 (Twice daily)	The response of drug was assessed for duration and severity of angina frequency, need of sublingual nitrate tablets	Over 6 months, 80% of patients reported subjective improvement, with a significant reduction in mean angina frequency (8.87 ± 7.18 to 3.03 ± 3.74, p < 0.001). Sublingual tablet consumption also decreased (17.37 ± 12.59 to 5.8 ± 5.79, p < 0.001). Among 11 hypertensive patients, five showed a non-significant drop in systolic blood pressure. Notably, 33.33% sustained improved exercise tolerance for 2 years	Free radical scavenging activity of MAK-4 and MAK-5 might decrease the amount of myocardial ischaemic injury Subjective and objective improvement in patient of angina suggests the role of MAK in long term effect in myocardial ischaemic diseases	Cardiovascular disease Angina pain	MAK-4 and MAK-5	Angina pectoris	Full paper	Dogra et al. (1994)
Clinical (Open-label)	Eighty patients with proven IHD Control arm (n = 40), MAK (n = 40)	10 g MAK-4 and 500 mg MAK-5 (Twice daily)	Clinical parameters like ejection fraction, mean angina frequency was assessed using echocardiography Investigation of plasm lipid peroxide was done	In 34 patients, the monthly occurrences of anginal episodes decreased from 5.50 (±3.20) to 2.15 (±2.00). Seven patients demonstrated enhanced ejection fraction during the treadmill test, and the plasma lipid peroxide levels dropped from 7.24 nmoles MDA/ml to 4.97 nmoles MDA/ml	Lowering of lipid peroxides suggested the prevention of atherogenesis and lowers the incidence of myocardial ischaemia	Antioxidant Angina pain	MAK-4 and MAK-5	Ischemic heart disease	Abstract	Dogra and Bhargava (2000)
Clinical (small pilot clinical investigations)	10 hyperlipidaemic patients aged between 21 and 65 years with total cholesterol >200bg/dl 18 weeks	10 g MAK-4 and 500 mg MAK-5 (Twice daily)	Assessing the resistance to LDL oxidation induced by Cu+2 and endothelial cell (EC) oxidation, measuring plasma lipid peroxide levels, and investigating lipid profiles (including apolipoprotein A and B, lipoprotein levels) following MAK intake	The lag phases for Cu+2-catalyzed LDL oxidation at baseline and after 6, 12, and 18 weeks of MAK-4 and MAK-5 intake were 6.66h ±0.19, 7.22h ±0.24 and 18 h ± 0.73 respectively. Endothelial cell-induced LDL oxidation showed lag phases of 14.89h ±0.77, 13.33h ±0.50, 20.22h ±0.76, and 20.00h ±0.79, respectively. Plasma lipid peroxide levels remained unchanged, with no notable alterations in plasma lipoproteins, apolipoprotein A, apolipoprotein B, and lipoprotein (a) levels	MAK-4 and MAK-5 inhibit LDL oxidation in patients with hyperlipidaemia. Therefore, MAK-4 and MAK-5 may be useful in the prevention and treatment of atherosclerosis	Atherosclerosis	​	Resistance to LDL oxidation	​	Sundaram et al. (1997)
Clinical (controlled, non-randomized multimodality intervention study)	173 Men and women mean age ≥65 years One group with MVM (with MAK intake) and the other with modern medicine	10 g MAK-4 and 500 mg MAK-5 (Twice daily)	Assessing the effectiveness of a 12-month, MVM-based multimodality intervention in promoting health and preventing disease in older individuals. Carotid intima-media thickness (IMT) was measured using B-mode ultrasound both before and after the year-long treatment	In the MVM group, 16 out of 20 subjects showed a decrease in carotid intima-media thickness (IMT), compared to a combined 12 out of 23 in the modern and usual care groups. This suggests that MVM may have the potential to counteract the effects of psychosocial and oxidative stress	Multimodality traditional approach can attenuate atherosclerosis in older subjects, particularly those with marked CHD risk Average decreases of 8% and 33% in the risk of acute myocardial infarction and stroke in the larger group and high-risk subgroup, respectively, of MVM subjects, relative to the modern and usual-care controls combined	Cardiovascular disease	MAK-4 and MAK-5	CHD and stroke	​	Fields et al. (2002)

A narrative synthesis approach was used to summarize the findings, identify patterns across studies, and highlight gaps in the current evidence base regarding the cardioprotective potential of MAK. This approach allowed a comprehensive mapping of available evidence and identification of areas requiring further investigation.

## 3 Results

**FIGURE 2:**
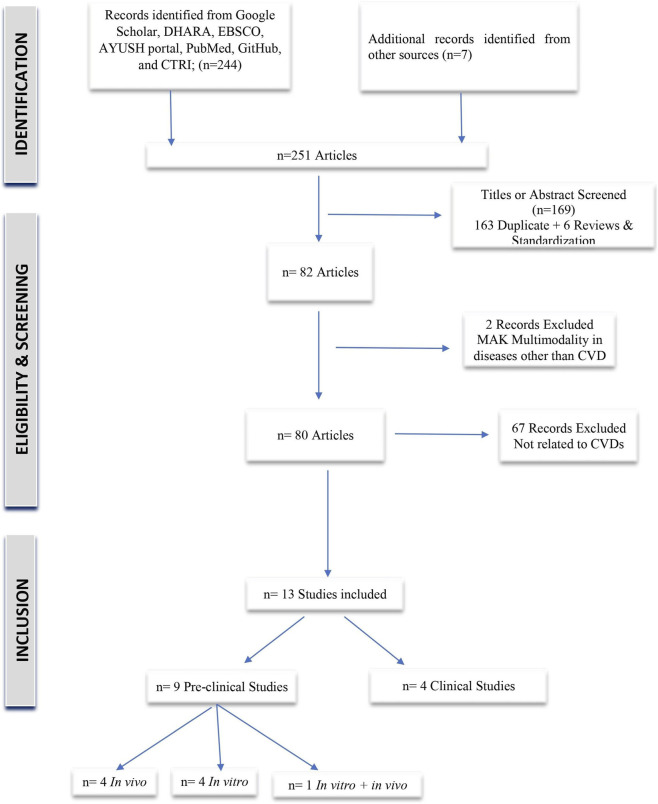
PRISMA flow diagram illustrating the study selection process for the scoping review.

Out of the 82 studies screened, 13 met the inclusion criteria for this review (Figure 2). Of these, nine were preclinical studies (four *in vitro* studies, four *in vivo* studies, and one hybrid study including both *in vitro* and *in vivo* methods), and four were clinical investigations. Most studies were conducted in the United States and India between 1988 and 2025. Findings are presented below according to the possible role of MAK in supporting heart health.

### 3.1 Evidence on MAK in oxidative stress and LDL oxidation

Preclinical studies consistently reported that MAK inhibits oxidative modifications of LDL, a key factor in atherosclerosis development. In isolated rat hearts, MAK-4 supplementation improved recovery of cardiac tension after hydrogen peroxide exposure, whereas control hearts exhibited contractile dysfunction throughout the 90-min testing period (Cullen et al., 1997).

Studies in WHHL rabbits demonstrated that MAK-4 delayed LDL oxidation induced by copper and endothelial cells. The lag phase for endothelial cell-induced LDL oxidation extended from 3 h in controls to 6 h in the MAK group, while copper-catalyzed oxidation showed no lag in controls compared to a 6-h lag in the MAK group (Hanna et al., 1995).

In hyperlipidaemic patients, MAK extracts inhibited LDL oxidation in a concentration-dependent manner. The IC50 for endothelial cell-induced LDL oxidation was 150.0 ± 10.0 nmol/μg (aqueous extract) and 69.3 ± 8.1 nmol/μg (ethanolic extract). For soybean lipoxygenase (SLP)-induced oxidation, IC50 values were 488.3 ± 41.9 nmol/μg and 128.3 ± 18.9 nmol/μg, respectively (Hanna et al., 1996). These findings indicate that MAK exhibits antioxidant activity that may reduce LDL susceptibility to oxidative modification.

### 3.2 Evidence on MAK in lipid peroxidation and cellular integrity

MAK was reported to inhibit both enzymatic and non-enzymatic lipid peroxidation. In WHHL rabbits, MAK-4 reduced the aortic plaque area from 47.6% ± 6.8% in controls to 22.5% ± 4.2% (p < 0.01) (Lee et al., 1996). Lipid peroxide levels and LDL/VLDL electrophoretic mobility were lower in MAK-treated animals, suggesting enhanced protection of lipoproteins (Lee et al., 1994).

Creatine kinase levels (CK, CK-BB, CK-MM) were also lower in the MAK group after cholesterol feeding, indicating preservation of myocardial integrity (Lee et al., 1994). *In vitro* studies with human LDL showed that both aqueous and alcoholic MAK extracts significantly reduced thiobarbituric acid-reactive substances (TBARS), reflecting inhibition of lipid peroxidation (Sharma et al., 1992). The alcoholic extract demonstrated markedly higher potency compared with standard antioxidants such as vitamin E or ascorbic acid after 6-h incubation.

### 3.3 Evidence on MAK and platelet aggregation and thrombosis risk

MAK demonstrated effects on platelet aggregation. Pre-incubation of platelet-rich plasma with MAK prior to exposure to agonists such as arachidonic acid, collagen, epinephrine, and ADP reduced aggregation in a dose-dependent manner. Complete reversal of ADP-induced aggregation and near-complete inhibition of collagen-induced aggregation were reported. In whole blood, 100% inhibition of platelet aggregation induced by collagen and 78% inhibition induced by ADP were reported (Sharma et al., 1989). These observations indicate that MAK may influence platelet aggregation pathways associated with thrombosis.

### 3.4 Evidence on MAK in atheroma formation and plaque development

Animal studies reported anti-atherogenic effects associated with MAK administration. WHHL rabbits receiving MAK-4 showed smaller atheromatous plaques (20.7% ± 3.7%) compared with controls (34.5% ± 5.2%) (Hanna et al., 1995). MAK administration was also associated with delayed LDL oxidation and reduced plasma peroxide levels.

These observations suggest that the combined antioxidant, lipid peroxidation-inhibiting, and platelet-modulating activities reported for MAK may be associated with slower plaque development and atheroma formation (Lee et al., 1996).

### 3.5 Clinical evidence on MAK in angina and ischemic heart disease

Clinical studies evaluated MAK in patients with angina and ischemic heart disease. In a study of 30 patients, Dogra et al. (1994) reported that 80% of participants experienced relief from angina. The mean monthly frequency of angina decreased from 8.87 ± 7.18 to 3.03 ± 3.74 (p < 0.001) after 6 months of combined MAK-4 and MAK-5 therapy. Angina duration decreased from 2.70 ± 4.50 min to 1.89 ± 4.30 min, and sublingual nitrate consumption declined from 17.37 ± 12.59 to 5.8 ± 5.79 per month (Dogra et al., 1994).

Dogra and Bhargava (2000) studied 80 patients with ischemic heart disease and reported a reduction in angina episodes from 5.50 ± 3.20 to 2.15 ± 2.00 per month. Ejection fraction improved, and lipid peroxide levels decreased from 7.24 to 4.97 nmoles MDA/ml, although changes in lipoprotein profiles were minimal.

Sundaram et al. (1997) conducted an 18-week trial in 10 hyperlipidaemic patients and observed time-dependent inhibition of LDL oxidation, with extension of the lag phase for both copper- and endothelial cell-induced LDL oxidation, reaching maximal effect at 18 weeks.

In another analysis, lag phases for Cu^2+^-catalyzed LDL oxidation were investigated at baseline and after 6, 12, and 18 weeks of consuming MAK-4 and MAK-5. The respective lag phases were 6.66h ±0.19, 7.22h ±0.24, and 18h ±0.73. Similarly, endothelial cell-induced LDL oxidation exhibited lag phases of 14.89h ±0.77, 13.33h ±0.50, 20.22h ±0.76, and 20.00h ±0.79 across the same time intervals. Throughout the study, plasma lipid peroxide levels remained stable, and no notable changes were observed in plasma lipoproteins, apolipoprotein A, apolipoprotein B, or lipoprotein(a) levels (Sundaram et al., 1997).

### 3.6 Evidence on MAK in vascular parameters in combined interventions

A multimodal study involving 173 participants (mean age ≥65 years) evaluated MAK supplementation in combination with transcendental meditation. The intervention was associated with reductions of up to 1 mm in intima–media thickness (IMT). Relative risk reductions of 8% for acute myocardial infarction and 33% for stroke were reported in high-risk subgroups compared with combined modern and usual-care controls (Fields et al., 2002).

Preclinical studies on MAK report several biological activities relevant to cardiovascular physiology, including antioxidant activity, inhibition of LDL oxidation, reduction of lipid peroxidation, modulation of platelet function, and attenuation of atheroma formation. Clinical investigations have reported outcomes such as changes in angina frequency and severity, ejection fraction, exercise tolerance, and vascular parameters.

Overall, the available literature indicates that MAK has been explored in relation to multiple cardiovascular-related mechanisms and clinical parameters. However, the evidence remains limited in scope and design, and further well-designed studies would be required to more clearly characterize these observations.

### 3.7 Safety assessments

Available studies have evaluated the toxicity profile of MAK. In one *in vitro* investigation, MAK-4 and MAK-5 showed mild cytotoxicity in the Rat-6 cell line (Dwivedi et al., 2005; Engineer et al., 1992). Across different experimental systems, including cell cultures, animal models, and human studies, MAK has generally been reported to be well tolerated. Clinical studies have also indicated good tolerability at doses ranging from 10 to 20 g per day, with no significant adverse effects reported.

## 4 Discussion

### 4.1 Overview of evidence

This scoping review systematically compiled preclinical and clinical studies examining MAK in relation to cardiovascular health. Most of the available clinical studies were exploratory in nature, typically open-label and non-randomized, with only one controlled pilot trial identified. The available literature reports several biological activities associated with MAK, including antioxidant activity, inhibition of LDL oxidation, reduction of lipid peroxidation, and modulation of platelet aggregation. Preclinical studies described these biochemical effects in experimental models such as rats and hyperlipidemic rabbits, while a limited number of clinical investigations reported outcomes including angina frequency, exercise tolerance, and markers related to arterial health.

Collectively, the studies indicate that MAK has been investigated in relation to multiple pathways relevant to cardiovascular physiology. However, the available evidence is limited in scope and methodological rigor, and therefore does not allow definitive conclusions regarding its clinical effectiveness in cardiovascular disease (CVD) management (Fields et al., 2002; Dogra and Bhargava, 2000; Cullen et al., 1997; Sundaram et al., 1997, 1995; Hanna et al., 1996; Lee et al., 1996; Dogra et al., 1994; Lee et al., 1994; Sharma et al., 1992, Sharma et al., 1989).

### 4.2 Mechanistic insights

The biological activities reported for MAK appear to arise from the combined actions of its multiple botanical drugs rather than a single dominant mechanism. As summarized in Table 1, the formulation contains several botanical drugs with documented antioxidant, anti-inflammatory, lipid-modulating, endothelial-supportive, and antithrombotic properties (Ullah et al., 2020; Alves-Silva et al., 2016; Kamath and Shah, 2015, 2014; Lakkur et al., 2015; Joshi and Bhonde, 2014; Ma et al., 2009). The integration of these reported activities aligns with the Rasayana concept in Ayurveda, which emphasizes systemic support rather than disease-specific targeting.

Rasayana therapy, a key component of Ayurveda and traditional, complementary, and integrative medicine, described as supporting physiological resilience and enhancing the body’s natural defense mechanisms (Bhinde, 2013). In Ayurvedic texts, Rasayana formulations are also described as strengthening *Ojas*, a concept associated with vitality and systemic balance (Lal et al., 2024; Bhendekar et al., 2023). MAK, derived from the classical Brahma Rasayana described in the Charaka Samhita, is intended in Ayurvedic practice to support systemic nourishment and balance, including the heart and mind (Lal et al., 2024).

Several botanical drugs of MAK, including *Emblica officinalis*, *Terminalia chebula*, and *Curcuma longa*, contain polyphenols and flavonoids that have been widely reported to exhibit free-radical scavenging activity and inhibition of lipid peroxidation (Gandhi et al., 2023; Sultan et al., 2023; Fuloria et al., 2022). These mechanisms are relevant to the oxidative modification of LDL, an early process involved in atherogenesis (Batty et al., 2022; Ullah et al., 2020; Lakkur et al., 2015).

Other botanical drugs such as *W. somnifera*, *Boerhaavia diffusa*, and *G. glabra* have been associated with modulation of inflammatory pathways and protection against oxidative tissue injury in experimental studies (Mikulska et al., 2023; Das et al., 2023; Pastorino et al., 2018). Similarly, botanical drugs including *C. asiatica*, *Piper longum*, and *Cinnamomum zeylanicum* have been reported to influence endothelial function, lipid metabolism, and platelet activity in experimental or pharmacological studies (Jagetia, 2021; Singh et al., 2021; Alok et al., 2013).

The presence of multiple botanical drugs with activities across these interconnected pathways provides a plausible explanation for the biochemical observations reported in experimental studies of MAK, including antioxidant activity, inhibition of LDL oxidation, and modulation of platelet aggregation (Fields et al., 2002; Dogra and Bhargava, 2000; Cullen et al., 1997; Lee et al., 1996; Hanna et al., 1996; Dogra et al., 1994; Sundaram et al., 1995; 1997). However, the relative contribution of individual constituents and their interactions within the formulation remain incompletely understood.

Experimental findings suggest that MAK-4 and MAK-5 possess antioxidant activity, attributed to the presence of flavonoids, alkaloids, terpenes, and polyphenolic metabolites (Kamath and Shah, 2015; 2014). *In vitro* studies report that MAK-4 scavenges hydrogen peroxide (H_2_O_2_), reducing its levels by approximately 50% (Sundaram et al., 1995). Both MAK-4 and MAK-5 have also been reported to inhibit LDL oxidation and lipid peroxidation in experimental systems, processes associated with oxidative stress and atherosclerotic development (Sundaram et al., 1997; 1995).

In addition, experimental studies have reported inhibitory effects of MAK on platelet aggregation induced by arachidonic acid, adenosine diphosphate (ADP), and collagen (Lebas et al., 2019; Greer et al., 1985). While these observations provide insight into potential mechanisms, their translation to human cardiovascular outcomes has not been fully established. Overall, the evidence summarized in Table 2 indicates that MAK has been investigated across multiple pathways relevant to cardiovascular physiology, including oxidative stress, lipid oxidation, and platelet activity (Singh et al., 2026). However, the clinical relevance of these mechanisms warrants further validation through well-designed human studies.

### 4.3 Clinical implications

The available clinical evidence indicates outcomes such as changes in angina frequency and duration, improvements in exercise tolerance, and variations in selected cardiovascular parameters, including ejection fraction and arterial thickness (Fields et al., 2002; Dogra and Bhargava, 2000; 1994; Sundaram et al., 1997). However, these studies were generally small and exploratory in design. No major adverse events were reported in the included studies, although systematic safety assessments were limited (Hanna et al., 1996; Rodella et al., 2004).

Given the limited number of studies and their methodological constraints, the findings should be interpreted cautiously. At present, the available evidence primarily indicates that MAK has been investigated in clinical contexts related to cardiovascular health rather than establishing its therapeutic efficacy.

### 4.4 Quality of evidence and potential biases

Although this review aimed to map the available evidence rather than formally assess study quality, several methodological limitations were apparent across the included studies. Many of the clinical investigations were small, open-label, and non-randomized, with limited reporting of allocation methods, blinding procedures, or control groups, increasing the potential for selection and performance bias. Outcome measures in several studies relied on subjective endpoints such as patient-reported angina frequency or exercise tolerance, which may introduce reporting bias. In addition, incomplete methodological reporting, particularly in older studies, limited the ability to evaluate risk of bias comprehensively. Preclinical studies also varied in experimental design, animal models, and outcome assessment methods, which may affect reproducibility and comparability. Collectively, these factors highlight the need for more rigorously designed experimental and clinical studies with standardized methodologies, appropriate controls, and transparent reporting to strengthen the evidence base surrounding MAK.

### 4.5 Conclusion

This scoping review maps the existing preclinical and clinical literature investigating MAK in relation to cardiovascular health. The studies describe several biological activities associated with MAK, including antioxidant activity, inhibition of LDL oxidation, and modulation of platelet aggregation. Clinical studies have explored outcomes such as angina symptoms, exercise tolerance, and selected cardiovascular parameters, although the evidence remains limited and heterogeneous.

By synthesizing and categorizing the available studies, this review highlights the current evidence base and identifies important gaps in knowledge. Further well-designed experimental and clinical investigations are required to clarify the mechanisms, safety profile, and clinical relevance of MAK in cardiovascular health.

## References

[B1] Almatroodi S. A. Alsahli M. A. Almatroudi A. Dev K. Rafat S. Verma A. K. (2020). Amla (*Emblica officinalis*): role in health management *via* controlling various biological activities. Gene Rep. 21, 100820. 10.1016/j.genrep.2020.100820

[B2] Alok S. Jain S. K. Verma A. Kumar M. Mahor A. Sabharwal M. (2013). Plant profile, phytochemistry and pharmacology of *Asparagus racemosus* (Shatavari): a review. Asian pac. J. Trop. Dis. 3 (3), 242–251. 10.1016/S2222-1808(13)60049-3

[B3] Alsaidan A. A. (2025). Cardiovascular disease management and prevention in Saudi Arabia: strategies, risk factors, and targeted interventions. Int. J. Clin. Pract. 2025, 7233591. 10.1155/ijcp/7233591

[B4] Alves-Silva J. M. Zuzarte M. Marques C. Salgueiro L. Girão H. (2016). Protective effects of terpenes on the cardiovascular system: current advances and future perspectives. Curr. Med. Chem. 23 (40), 4559–4600. 10.2174/0929867323666160907123559 27604093

[B5] Arksey H. O’Malley L. (2005). Scoping studies: towards a methodological framework. Int. J. Soc. Res. Methodol. 8 (1), 19–32. 10.1080/1364557032000119616

[B135] Ashokkumar K. Murugan M. Dhanya M. K. Warkentin T. D. (2020). Botany, traditional uses, phytochemistry and biological activities of cardamom (Elettaria cardamomum (L.) Maton) – a critical review. J. Ethnopharmacol. 246, 112244. 10.1016/j.jep.2019.112244 31541721

[B6] Ashwini M. B. Seema B. Medikeri S. S. (2022). Review on Ikshu and its vikaras. J. Ayu. Integr. Med. Sci. 7 (1), 295–303. Available online at: https://jaims.in/jaims/article/view/1686

[B7] Balkrishna A. Thakur P. Varshney A. (2020). Phytochemical profile, pharmacological attributes and medicinal properties of *Convolvulus prostratus*: a cognitive enhancer herb for the management of neurodegenerative etiologies. Front. Pharmacol. 11, 171. 10.3389/fphar.2020.00171 32194410 PMC7063970

[B8] Balkrishna A. Agarwal U. Saxena S. Sharma G. Arya V. (2026). Toward an integrated therapeutic approach for familial hypercholesterolemia. Curr. Med. Sci. 46, 65–78. 10.1007/s11596-025-00149-6 41511735

[B9] Batty M. Bennett M. R. Yu E. (2022). The role of oxidative stress in atherosclerosis. Cells 11 (23), 3843. 10.3390/cells11233843 36497101 PMC9735601

[B10] Bharti R. Chopra B. S. Raut S. Khatri N. (2021). *Pueraria tuberosa*: traditional uses, pharmacology, and phytochemistry. Front. Pharmacol. 11, 582506. 10.3389/fphar.2020.582506 33708108 PMC7941752

[B11] Bhati M. Sharma B. Mishra P. K. Choudhary B. P. (2025). An Ayurvedic concept of Rasayana therapy for geriatric wellness and longevity. World J. Pharm. Res. 14 (18), 47–61. 10.20959/wjpr202518-38191

[B12] Bhendekar M. V. Katekar V. A. Deshmukh S. P. (2023). Ayurveda’s role in preventing and managing cardiovascular diseases: a comprehensive review. GSC Biol. Pharm. Sci. 25 (2), 141–148. 10.30574/gscbps.2023.25.2.0420

[B13] Bhinde S. (2013). Rasayana: a better alternative for disease prevention. J. Ayurveda Holist. Med. 1 (9), 6–14. 10.70066/jahm.v1i9.137

[B14] Biswas P. Ghorai M. Mishra T. Gopalakrishnan A. V. Roy D. Mane A. B. (2022). *Piper longum* L.: traditional uses, phytochemistry, pharmacology, and health-promoting activities. Phytother. Res. 36 (12), 4425–4476. 10.1002/ptr.7649 36256521

[B15] Bondy S. C. Hernandez T. M. Mattia C. (1994). Antioxidant properties of two ayurvedic herbal preparations. Biochem. Arch. 10 (1), 25–31.

[B16] Boughaleb H. Verdoy R. Pochet A. Fabian N. Bella R. Muruganandam G. (2025). Repurposing *Bacopa monnieri* extracts containing aquaporin-1 blockers to improve systemic oxidative stress: the bacoxy_i study. Adv. Redox Res. 15, 100126. 10.1016/j.arres.2025.100126

[B17] Caturano A. Morciano C. Zielińska K. Russo V. Perrone M. A. Berra C. C. (2025). Rethinking the diabetes–cardiovascular disease continuum: toward integrated care. J. Clin. Med. 14 (18), 6678. 10.3390/jcm14186678 41010881 PMC12471255

[B18] Centers for Disease Control and Prevention (2018). “Heart diseases,” in National Center for chronic disease prevention and health promotion.

[B19] Chauhan A. Semwal D. K. Mishra S. P. Semwal R. B. (2015). Ayurvedic research and methodology: present status and future strategies. Ayu 36 (4), 364–369. 10.4103/0974-8520.190699 27833362 PMC5041382

[B20] Choudhary S. Chaudhary G. (2021). Sandalwood (*Santalum album*): ancient tree with significant medicinal benefits. Int. J. Ayurveda Pharm. Res. 9 (4), 90–99. 10.47070/ijapr.v9i4.1895

[B21] Cullen W. Dulchavsky S. Devasagayam T. Venkataraman B. Dutta S. (1997). Effect of Maharishi MAK-4 on H2O2-induced oxidative stress in isolated rat hearts. J. Ethnopharmacol. 56 (3), 215–222. 10.1016/s0378-8741(97)01526-2 9201611

[B22] Das S. Singh P. K. Ameeruddin S. Kumar Bindhani B. Obaidullah W. J. Obaidullah A. J. (2023). Ethnomedicinal values of *Boerhaavia diffusa* L. as a panacea against multiple human ailments. Front. Chem. 11, 1297300. 10.3389/fchem.2023.1297300 38033469 PMC10682173

[B23] Dogra J. Bhargava A. (2000). Lipid peroxide in ischemic heart disease: inhibition by Maharishi Amrit Kalash (MAK-4 and MAK-5) herbal mixtures (Abstract). FASEB J. 14 (4), A121.

[B24] Dogra J. Grover N. Kumar P. Aneja N. (1994). Indigenous free radical scavenger MAK-4 and MAK-5 in angina pectoris: is it only a placebo? J. Assoc. Physicians India 42 (6), 466–467. 7852231

[B25] Dwivedi C. Satter B. Sharma H. (1988). Anticarcinogenic activity of an ayurvedic food supplement, Maharishi Amrit Kalash (AK). Am. Physiol. Soc.ASPET Proc. 30, A121.

[B26] Dwivedi C. Agarwal P. Natarajan K. Sharma H. (2005). Antioxidant and protective effects of Amrit Nectar tablets on adriamycin- and cisplatin-induced toxicities. J. Altern. Complement. Med. 11 (1), 143–148. 10.1089/acm.2005.11.143 15750373

[B27] Ekor M. (2014). The growing use of herbal medicines: issues relating to adverse reactions and challenges in monitoring safety. Front. Pharmacol. 4, 177. 10.3389/fphar.2013.00177 24454289 PMC3887317

[B28] Engineer F. N. Sharma H. Dwivedi C. (1992). Protective effects of M-4 and M-5 on adriamycin-induced microsomal lipid peroxidation and mortality. Biochem. Arch. 8, 267–272.

[B29] Fields J. Walton K. Schneider R. Nidich S. Pomerantz R. Suchdev P. (2002). Effect of a multimodality natural medicine program on carotid atherosclerosis in older subjects: a pilot trial of Maharishi Vedic medicine. Am. J. Cardiol. 89 (8), 952–958. 10.1016/S0002-9149(02)02245-2 11950434

[B30] Floria M. Trifan A. V. Tanase D. M. (2026). The cardiovascular disease continuum: from cardiovascular risk factors to heart failure. Life 16 (2), 308. 10.3390/life16020308 41752944 PMC12942550

[B31] Fuloria S. Mehta J. Chandel A. Sekar M. Rani N. N. I. M. Begum M. Y. (2022). Therapeutic potential of *Curcuma longa* Linn. in relation to its major active constituent curcumin. Front. Pharmacol. 13, 820806. 10.3389/fphar.2022.820806 35401176 PMC8990857

[B32] Fursule R. A. Patil S. D. (2010). Hepatoprotective and antioxidant activity of *Phaseolus trilobus* Ait. on bile duct ligation-induced liver fibrosis in rats. J. Ethnopharmacol. 129 (3), 416–419. 10.1016/j.jep.2010.04.021 20430092

[B33] Galani V. J. Patel B. G. Patel N. B. (2010). *Argyreia speciosa* (Linn. f.) Sweet: a comprehensive review. Pharmacogn. Rev. 4 (8), 172–178. 10.4103/0973-7847.70913 22228958 PMC3249918

[B34] Gandhi Y. Grewal J. Jain V. Rawat H. Mishra S. K. Kumar V. (2023). *Emblica officinalis*: a promising herb confining versatile applications. S. Afr. J. Bot. 159, 519–531. 10.1016/j.sajb.2023.06.041

[B35] Gelderloos P. Ahlstrom H. H. B. Orme-Johnson D. W. Robinson D. K. Wallace R. K. Glaser J. L. (1990). Influence of a Maharishi Ayur-Vedic herbal preparation on age-related visual discrimination. Int. J. Psychosom. 37 (1–4), 25–29. 2246098

[B36] Ghoshal A. Sharma P. Sudhakaran D. Dutta E. Connor S. Ali Z. (2026). Integrating palliative care into the noncommunicable disease framework in low- and middle-income countries. Adv. Public Health 2026, 5520274. 10.1155/adph/5520274

[B37] Glaser J. L. (1988). Maharishi Ayurveda: an introduction to recent research. Mod. Sci. Ved. Sci. 2, 89–108.

[B38] Government of India (2003). The ayurvedic formulary of India. 2nd ed. New Delhi: Ministry of Health and Family Welfare, 43.

[B39] Goyal M. (2018). Rasayana in perspective of the present scenario. Ayu 39 (2), 63–64. 10.4103/ayu.AYU_300_18 30783358 PMC6369608

[B40] Greer I. Walker J. Calder A. Forbes C. (1985). Inhibition of platelet aggregation in whole blood by adrenoceptor antagonists. Thromb. Res. 40 (5), 631–643. 10.1016/0049-3848(85)90301-9 3937284

[B136] Gupta R. Mehta M. R. (2026). Scientific integration of complementary and alternative medicine with modern medicine for enhancing patient outcomes through holistic care. J. Mod. Med. 4 (1), 4–8. 10.4103/JOMM.JOMM_8_25

[B41] Hafez L. O. Brito-Casillas Y. Abdelmageed N. Alemán-Cabrera I. M. Morad S. A. F. Abdel-Raheem M. H. (2024). *Vachellia nilotica*: traditional uses and recent advances on pharmacological attributes. Nutrients 16 (24), 4278. 10.3390/nu16244278 39770900 PMC11678605

[B42] Hanna A. Lee J. Kauffman E. Lott J. Sharma H. (1995). Antiatherogenic properties of the herbal mixture MAK-4 in Watanabe heritable hyperlipidemic rabbits (Abstract). FASEB J. 9 (3), A141.

[B43] Hanna A. Sundaram V. Falko J. Stephens R. Sharma H. (1996). Effect of herbal mixtures MAK-4 and MAK-5 on susceptibility of human LDL to oxidation. Complement. Med. Int. 3 (3), 28–36.

[B44] Harminder V. Singh V. Chaudhary A. K. (2011). Taxonomy, ethnobotany, chemistry and pharmacology of *Oroxylum indicum* vent. Indian J. Pharm. Sci. 73 (5), 483–490. 10.4103/0250-474X.98981 22923859 PMC3425058

[B134] Hassan A. Malik K. Naqvi S. A. M. Khan K. Sadia H. (2024). A comprehensive review of Saccharum spontaneum, its traditional uses, phytochemistry and pharmacologys. Ethnobot. Res. Appl. 29, 1–13. Available online at: https://ethnobotanyjournal.org/index.php/era/article/view/5918.

[B133] Hasan F. Gaur P. K. Thippamma K. Shahreen N. Talebi M. (2025). A comprehensive overview of phytochemical composition and therapeutic applications of Sida cordifolia L. PhytoMed. Plus 5 (03), 100856. 10.1016/j.phyplu.2025.100856

[B45] Inaba R. Sugiura H. Iwata H. (1995). Immunomodulatory effects of Maharishi Amrit Kalash 4 and 5 in mice. Jpn. J. Hyg. 50 (4), 901–905. 10.1265/jjh.50.901 8538064

[B46] Inaba R. Sugiura H. Iwata H. Mori H. Tanaka T. (1996). Immunomodulation by Maharishi Amrit Kalash 4 in mice. J. Appl. Nutr. 48 (1–2), 10–21.

[B47] Inaba R. Sugiura H. Iwata H. Tanaka T. (1997). Dose-dependent activation of immune function in mice by ingestion of Maharishi Amrit Kalash 5. Environ. Health Prev. Med. 2 (1), 35–39. 10.1007/BF02931227 21432448 PMC2723328

[B48] Iqubal M. Sharma S. K. Hussain M. S. Mujahid M. (2022). An updated ethnobotany, phytochemical and pharmacological potential of Solanum indicum L. J. Drug Deliv. Ther. 12 (2), 160–172. 10.22270/jddt.v12i2.5385

[B49] Iweala E. J. Uche M. E. Dike E. Etumnu L. R. Dokunmu T. M. Oluwapelumi A. E. (2023). Curcuma longa (Turmeric): ethnomedicinal uses, phytochemistry, pharmacological activities and toxicity profiles—A review. Pharmacol. Res. Mod. Chin. Med. 6, 100222. 10.1016/j.prmcm.2023.100222

[B50] Jagetia G. C. (2021). A review on the medicinal and pharmacological properties of traditional ethnomedicinal plant Sonapatha, Oroxylum indicum. Sinusitis 5 (1), 71–89. 10.3390/sinusitis5010009

[B51] Jan B. Dar M. I. Choudhary B. Basist P. Khan R. Alhalmi A. (2024). Cardiovascular diseases among Indian older adults: a comprehensive review. Cardiovasc. Ther. 2024, 6894693. 10.1155/2024/6894693 39742010 PMC11323990

[B52] Jena J. Gupta A. K. (2012). Ricinus communis Linn.: a phytopharmacological review. Int. J. Pharm. Pharm. Sci. 4 (4), 25–29.

[B53] Jiang H. Zhou Y. Nabavi S. M. Sahebkar A. Little P. J. Xu S. (2022). Mechanisms of oxidized LDL-mediated endothelial dysfunction and its consequences for the development of atherosclerosis. Front. Cardiovasc. Med. 9, 925923. 10.3389/fcvm.2022.925923 35722128 PMC9199460

[B54] Jiao X. Guo D. Li W. Li S. Shao G. Shen T. (2025). Foam cells: tracing the diverse cellular origins and their linked signaling pathways. J. Transl. Med. 23, 1382. 10.1186/s12967-025-07402-5 41351176 PMC12681170

[B55] Joshi K. S. Bhonde R. (2014). Insights from Ayurveda for translational stem cell research. J. Ayurveda Integr. Med. 5 (1), 4–10. 10.4103/0975-9476.128846 24812469 PMC4012361

[B56] Joshi S. Kumar S. (2024). “A review of some medicinal grasses from Ayurveda,” in Medicinal Poaceae of India, 1.

[B57] Kamath C. Shah B. (2014). Phytochemical screening and standardization of polyherbal formulation: Maharishi Amrit Kalash 5. Int. J. Pharm. Pharm. Sci. 6 (7), 96–98. Available online at: https://journals.innovareacademics.in/index.php/ijpps/article/view/1268.

[B58] Kamath C. Shah B. (2015). Quantitative estimation of catechin, quercetin and ß-carotene from polyherbal formulation. Int. J. Pharm. Sci. Res. 6 (4), 1596–1601. 10.13040/IJPSR.0975-8232.6(4).1596-01

[B59] Koch A. K. Patel M. Gupta S. Wullenkord R. Jeitler M. Kessler C. S. (2024). Efficacy and safety of the Ayurvedic herbal preparation Maharishi Amrit Kalash: a systematic review of randomized controlled trials. Front. Med. (Lausanne). 11, 1325037. 10.3389/fmed.2024.1325037 38690176 PMC11058942

[B60] Kuerban M. Ma F. Shan L. Wang Y. Zhou G. (2024). Comparative discriminant analysis of Mesua ferrea L. and its adulterants. Heliyon 10 (7), e28459. 10.1016/j.heliyon.2024.e28459 38601576 PMC11004703

[B61] Lade S. Alok S. Nagdev S. Golani P. Agrawal A. Kosey S. (2022). A potential role of Stereospermum suaveolens DC for multiple diseases: a brief analysis. Int. J. Pharm. Sci. Res. 13 (9), 3486–3493. 10.13040/IJPSR.0975-8232.13(9).3486-93

[B62] Lakkur S. Judd S. Bostick R. M. McClellan W. Flanders W. D. Stevens V. L. (2015). Oxidative stress, inflammation, and markers of cardiovascular health. Atherosclerosis 243 (1), 38–43. 10.1016/j.atherosclerosis.2015.08.032 26343870 PMC4609620

[B63] Lal D. Mishra V. Pathak P. C. Singh V. B. (2024). Conceptual study of heart disease and its management: an ayurvedic perspective. Int. J. Ayurveda Pharma Res. 12 (2), 133–136. Available online at: https://ijapr.in/index.php/ijapr/article/view/3037 .

[B64] Lebas H. Yahiaoui K. Martos R. Boulaftali Y. (2019). Platelets are at the nexus of vascular diseases. Front. Cardiovasc. Med. 6, 132. 10.3389/fcvm.2019.00132 31572732 PMC6749018

[B65] Lee J. Y. Lott J. A. Sharma H. M. (1994). Biochemical changes induced by Maharishi Amrit Kalash (MAK-4) and MA-208 in diet-induced hypercholesterolemic rabbits. Adv. Exp. Med. Biol. 366, 446–447. 10.1007/978-1-4615-1833-4_48 7771288

[B66] Lee J. Y. Hanna A. N. Lott J. A. Sharma H. M. (1996). The antioxidant and antiatherogenic effects of MAK-4 in WHHL rabbits. J. Altern. Complement. Med. 2 (4), 463–478. 10.1089/acm.1996.2.463 9395676

[B67] Levac D. Colquhoun H. O'Brien K. K. (2010). Scoping studies: advancing the methodology. Implement. Sci. 5, 69. 10.1186/1748-5908-5-69 20854677 PMC2954944

[B68] Lin H. Wang W. Peng M. Kong Y. Zhang X. Wei X. (2024). Pharmacological properties of Polygonatum and its active ingredients for the prevention and treatment of cardiovascular diseases. Chin. Med. 19 (1), 1. 10.1186/s13020-023-00871-0 38163901 PMC10759625

[B69] Ma X. H. Zheng C. J. Han L. Y. Xie B. Jia J. Cao Z. W. (2009). Synergistic therapeutic actions of herbal ingredients and their mechanisms from molecular interaction and network perspectives. Drug Discov. Today. 14 (11-12), 579–588. 10.1016/j.drudis.2009.03.012 19508920

[B70] Mak S. Thomas A. (2022). Steps for conducting a scoping review. J. Grad. Med. Educ. 14 (5), 565–567. 10.4300/JGME-D-22-00621.1 36274762 PMC9580325

[B71] Mikulska P. Malinowska M. Ignacyk M. Szustowski P. Nowak J. Pesta K. (2023). Ashwagandha (Withania somnifera)-Current research on the health-promoting activities: a narrative review. Pharmaceutics 15 (4), 1057. 10.3390/pharmaceutics15041057 37111543 PMC10147008

[B72] Mohan Maruga Raja M. K. Mishra S. H. (2010). Comprehensive review of *Clerodendrum phlomidis*: a traditionally used bitter. Zhong Xi Yi Jie He Xue Bao 8 (6), 510–524. 10.3736/jcim20100602 20550872

[B73] Mohanty S. K. Swamy M. K. Sinniah U. R. Anuradha M. (2017). *Leptadenia reticulata* (Retz.) Wight & Arn. (Jivanti): botanical, agronomical, phytochemical, pharmacological, and biotechnological aspects. Molecules 22 (6), 1019. 10.3390/molecules22061019 28629185 PMC6152761

[B74] Monika S. Thirumal M. Kumar P. R. (2023). Phytochemical and biological review of Aegle marmelos Linn. Future Sci. OA 9 (3), FSO849. 10.2144/fsoa-2022-0068 37026028 PMC10072075

[B75] Mukherjee P. W. (2012). Quality control of herbal drugs: an approach to evaluation of botanicals. New Delhi, India: Business Horizons Publishers.

[B76] Munn Z. Peters M. D. J. Stern C. Tufanaru C. McArthur A. Aromataris E. (2018). Systematic review or scoping review? Guidance for authors when choosing between a systematic or scoping review approach. BMC Med. Res. Methodol. 18, 143. 10.1186/s12874-018-0611-x 30453902 PMC6245623

[B77] Namas R. Al Qassimi S. Almarzooqi A. (2025). Epidemiological perspectives on SLE and APS in MENA: burden, barriers, and a path toward improved outcomes. Lupus 35 (2), 208–217. 10.1177/09612033251408525 41419792

[B78] Nazar S. Hussain M. A. Khan A. Muhammad G. Tahir M. N. (2020). Capparis decidua Edgew. (Forssk.): a comprehensive review of its traditional uses, phytochemistry, pharmacology and nutrapharmaceutical potential. Arab. J. Chem. 13 (1), 1901–1916. 10.1016/j.arabjc.2018.02.007

[B79] Niharika R. Parveen R. Parveen B. Umar S. Ahmad S. (2026). A comprehensive review on indigenous therapeutic approaches in kidney care using Ayush medicine. Front. Pharmacol. 16, 1588424. 10.3389/fphar.2025.1588424 41695991 PMC12903127

[B80] Pastorino G. Cornara L. Soares S. Rodrigues F. Oliveira M. B. P. P. (2018). Liquorice (Glycyrrhiza glabra): a phytochemical and pharmacological review. Phytother. Res. 32 (12), 2323–2339. 10.1002/ptr.6178 30117204 PMC7167772

[B81] Patel V. K. Wang J. Shen R. N. Sharma H. M. Brahmi Z. (1992). Reduction of metastases of Lewis lung carcinoma by an ayurvedic food supplement in mice. Nutr. Res. 12 (4–5), 667–676. 10.1016/S0271-5317(05)80036-3

[B82] Pawar R. P. Nikam P. D. Waghmode A. V. Shinde H. P. (2023). Review on ecological and pharmacological aspects of Chlorophytum borivilianum Sant. and Fern. Sustain. Agri, Food Environ. Res. 12 (1), 1–11. 10.7770/safer-V12N1-art704

[B83] Penza M. Montani C. Jeremic M. Mazzoleni G. Hsiao W. L. W. Marra M. (2007). MAK-4 and -5 supplemented diet inhibits liver carcinogenesis in mice. BMC Complement. Altern. Med. 7, 19. 10.1186/1472-6882-7-19 17559639 PMC1894988

[B84] Perveen S. Khan M. A. Parveen R. Insaf A. Parveen B. Ahmad S. (2023). An updated review on traditional and modern aspects of Vitex negundo. Curr. Tradit. Med. 9 (2), e270822208079. 10.2174/2215083808666220827115915

[B85] Peters M. D. J. Marnie C. Tricco A. C. Pollock D. Munn Z. Alexander L. (2020). Updated methodological guidance for the conduct of scoping reviews. JBI Evid. Synth. 18 (10), 2119–2126. 10.11124/jbies-20-00167 33038124

[B86] Pirzada A. M. Ali H. H. Naeem M. Latif M. Bukhari A. H. Tanveer A. (2015). Cyperus rotundus L.: traditional uses, phytochemistry, and pharmacological activities. J. Ethnopharmacol. 174, 540–560. 10.1016/j.jep.2015.08.012 26297840

[B87] Prasad M. L. Parry P. Chan C. (1993). Ayurvedic agents produce differential effects on murine and human melanoma cells *in vitro* . Nutr. Cancer. 20 (1), 79–86. 10.1080/01635589309514273 8415133

[B88] Rakshe R. S. Kale A. Y. Otari K. V. Bhamkar S. J. Borade M. A. (2024). A comprehensive review of the medicinal properties of Teramnus labialis seeds: pharmacology, pharmacognosy, phytochemistry. Int. J. Creat. Res. Thoughts (IJCRT). 12 (8), 2320–2882.

[B89] Ramadevi S. Kaleeswaran B. Ilavenil S. Upgade A. Tamilvendan D. Rajakrishnan R. (2020). Effect of traditionally used herb Pedalium murex L. and its active compound pedalitin on urease expression - for the management of kidney stone. Saudi J. Biol. Sci. 27 (3), 833–839. 10.1016/j.sjbs.2020.01.014 32127759 PMC7042614

[B90] Rastogi S. Pandey M. M. Rawat A. K. (2011). An ethnomedicinal, phytochemical and pharmacological profile of Desmodium gangeticum (L.) DC. and Desmodium adscendens (Sw.) DC. J. Ethnopharmacol. 136 (2), 283–296. 10.1016/j.jep.2011.04.031 21530632

[B91] Rauniyar N. Srivastava D. (2020). *Ipomoea digitata*: a therapeutic boon from nature to mankind. J. Indian Bot. Soc. 100 (3–4), 185–191. 10.5958/2455-7218.2020.00039.x

[B92] Rodella L. Borsani E. Rezzani R. Lanzi R. Lonati C. Bianchi R. (2004). MAK-5 treatment enhances the nerve growth factor-mediated neurite outgrowth in PC12 cells. J. Ethnopharmacol. 93 (2–3), 161–166. 10.1016/j.jep.2003.12.033 15234748

[B93] Samaiya A. Srivastava A. Taranikanti V. Coshic O. H. Parshad R. Valthalure S. (1999). Reduction in toxicity of cancer chemotherapy by Maharishi Amrit Kalash (MAK)- an ayurvedic herbal compound. Ann. Natl. Acad. Med. Sci. (India) 35 (2), 109–119.

[B94] Saxena A. Dixit S. Aggarwal S. Vuthaluru S. Prashad R. Bhushan S. (2008). An ayurvedic herbal compound to reduce toxicity to cancer chemotherapy: a randomized controlled trial. Indian J. Med. Paediatr. Oncol. 29 (2), 11–18. 10.4103/0971-5851.51426

[B95] Scartezzini P. Speroni E. (2000). Review on some plants of Indian traditional medicine with antioxidant activity. J. Ethnopharmacol. 71, 23–43. 10.1016/s0378-8741(00)00213-0 10904144

[B96] Sharma P. V. (1983). Chikitsasthana (Varanasi: Chaukhamba Orientalia), 2, 118.

[B97] Sharma H. M. Feng Y. Panganamala R. V. (1989). Maharishi Amrit Kalash (MAK-5) prevents human Platelet aggregation. Clin. Terapia Cardiovasc. 8 (3), 227–230.

[B98] Sharma H. M. Krieger J. Dwivedi C. (1990). Antineoplastic properties of dietary Maharishi Amrit Kalash-4 and Maharishi Amrit Kalash-5, Ayurvedic food supplements. Eur. J. Pharmacol. 183 (2), 193. 10.1016/0014-2999(90)93027-N

[B99] Sharma H. M. Hanna A. N. Kauffman E. M. Newman H. A. (1992). Inhibition of human low-density lipoprotein oxidation *in vitro* by Maharishi Ayur-Veda herbal mixtures. Pharmacol. Biochem. Behav. 43 (4), 1175–1182. 10.1016/0091-3057(92)90500-f 1475302

[B100] Sharma H. M. Hanna A. N. Titterington L. C. Stephens R. E. (1994). Effect of MAK-4 and MAK-5 on endothelial cell and soybean lipoxygenase-induced LDL oxidation. Adv. Exp. Med. Biol. 366, 441–443. 10.1007/978-1-4615-1833-4_46 7771286

[B101] Sharma H. M. Lee J. Y. Kauffman E. M. Hanna A. N. (1996). *In vivo* effect of herbal mixture MAK-4 on antioxidant capacity of brain microsomes. Biochem. Arch. 12 (3), 181–186.

[B102] Sharma V. Hem K. Seth A. Maurya S. K. (2017). Solanum indicum Linn: an ethnopharmacological, phytochemical and pharmacological review. Curr. Res. J. Pharm. Allied Sci. 1 (2), 1–9.

[B103] Sharma R. Singla R. K. Banerjee S. Sinha B. Shen B. Sharma R. (2022). Role of Shankhpushpi (Convolvulus pluricaulis) in neurological disorders: an umbrella review covering evidence from ethnopharmacology to clinical studies. Neurosci. Biobehav. Rev. 140, 104795. 10.1016/j.neubiorev.2022.104795 35878793

[B104] Sharma V. Gautam D. N. S. Radu A. F. Behl T. Bungau S. G. Vesa C. M. (2022). Reviewing the Traditional/Modern uses, phytochemistry, essential Oils/Extracts and pharmacology of *Embelia ribes* Burm. Antioxidants 11 (7), 1359. 10.3390/antiox11071359 35883850 PMC9311956

[B105] Shastri K. (1975). “Charak Samhita Chikitisasthana,”, 7. Varanasi: Chaukhambha Bharti Academy, 1.

[B106] Siddiqui S. Jadaun S. Anusha R. Kumar N. Ashraf S. A. Bishnoi J. P. (2025). Medicinal importance of Tinospora cordifolia (Willd.) Miers and its possible use in food industry for value addition. Discov. Food 5, 357. 10.1007/s44187-025-00652-8

[B107] Singh N. Rao A. S. Nandal A. Kumar S. Yadav S. S. Ganaie S. A. (2021). Phytochemical and pharmacological review of Cinnamomum verum J. Presl-a versatile spice used in food and nutrition. Food Chem. 338, 127773. 10.1016/j.foodchem.2020.127773 32829297

[B108] Singh R. Kaushik N. Vohra R. Shrivastava R. (2025). Immunomodulatory action of an ayurvedic polyherbal Formulation Maharishi Amrit kalash-scoping review. Indo. Glob. J. Pharm. Sci. 15, 1–15. 10.35652/IGJPS.2025.15001

[B109] Singh D. D. Yadav D. K. Shin D. (2026). Next-Generation antioxidants in cardiovascular disease: mechanistic insights and emerging therapeutic strategies. Antioxidants 15 (2), 164. 10.3390/antiox15020164 41750545 PMC12938358

[B110] Srivastava D. (2020). Ipomoea digitata: therapeutic boon from nature to mankind. J. Indian Bot. Soc. 99 (3), 1–7. 10.5958/2455-7218.200.00039.X

[B111] Ștefănescu R. Tero-Vescan A. Negroiu A. Aurică E. Vari C.-E. (2020). A comprehensive review of the phytochemical, pharmacological, and toxicological properties of Tribulus terrestris L. Biomolecules 10 (5), 752. 10.3390/biom10050752 32408715 PMC7277861

[B112] Sultan M. T. Anwar M. J. Imran M. Khalil I. Saeed F. Neelum S. (2023). Phytochemical profile and pro-healthy properties of Terminalia chebula: a comprehensive review. Int. J. Food Prop. 26 (1), 526–551. 10.1080/10942912.2023.2166951

[B113] Sundaram V. Hanna A. Lubow G. Falko J. Sharma H. (1995). Increased resistance of human LDL to oxidation in hyperlipidemic patients supplemented with oral herbal mixture MAK-4. FASEB J. 9 (3), A141.

[B114] Sundaram V. Hanna A. Lubow G. Koneru L. Falko J. Sharma H. (1997). Inhibition of low-density lipoprotein oxidation by oral herbal mixtures Maharishi Amrit Kalash-4 and Maharishi Amrit Kalash-5 in hyperlipidemic patients. Am. J. Med. Sci. 314 (5), 303–310. 10.1097/00000441-199711000-00007 9365332

[B115] Teo K. K. Rafiq T. (2021). Cardiovascular risk factors and prevention: a perspective from developing countries. Can. J. Cardiol. 37 (5), 733–743. 10.1016/j.cjca.2021.02.009 33610690

[B116] Tricco A. Lillie E. Zarin W. O'Brien K. K. Colquhoun H. Levac D. (2018). PRISMA extension for scoping reviews (PRISMA-ScR): checklist and explanation. Ann. Intern. Med. 169 (7), 467–473. 10.7326/M18-0850 30178033

[B117] Ullah A. Munir S. Badshah S. L. Khan N. Ghani L. Poulson B. G. (2020). Important flavonoids and their role as a therapeutic agent. Molecules 25 (22), 5243. 10.3390/molecules25225243 33187049 PMC7697716

[B118] Upadhyay S. Dixit M. (2015). Role of polyphenols and other phytochemicals on molecular signaling. Oxid. Med. Cell Longev. 2015, 1–15. 10.1155/2015/504253 26180591 PMC4477245

[B119] Vats S. Kaushal C. Timko M. P. Ganie S. A. (2024). Uraria picta: a review on its ethnobotany, bioactive compounds, pharmacology and commercial relevance. S. Afr. J. Bot. 167, 333–354. 10.1016/j.sajb.2024.02.008

[B120] Vilahur G. Fuster V. (2025). Interplay between platelets and coagulation: from protective haemostasis to pathological arterial thrombosis. Eur. Heart Journal 46 (5), 413–423. 10.1093/eurheartj/ehae776 39673717

[B121] Vohra B. P. S. Sharma S. P. Kansal V. K. (1999). Maharishi Amrit Kalash rejuvenates ageing central nervous system’s antioxidant defence system: an *in vivo* study. Pharmacol. Res. 40 (6), 497–502. 10.1006/phrs.1999.0540 10660948

[B122] Vohra B. Sharma S. Kansal V. (2001). Effect of Maharishi Amrit Kalash on age dependent variations in mitochondrial antioxidant enzymes, lipid peroxidation and mitochondrial population in different regions of the central nervous system of Guinea pigs. Drug Metab. Pharmacokinet. 18 (1), 57–68. 10.1515/dmdi.2001.18.1.57 11522125

[B123] Vohra R. Singh R. Shrivastava R. (2024). A scoping review on 'maharishi Amrit Kalash', an ayurveda formulation for cancer prevention and management. J. Ayurveda Integr. Med. 15 (1), 100866. 10.1016/j.jaim.2023.100866 38194855 PMC10792650

[B124] Wahab S. Annadurai S. Abullais S. S. Das G. Ahmad W. Ahmad M. F. (2021). Glycyrrhiza glabra (Licorice): a comprehensive review on its phytochemistry, biological activities, clinical evidence and toxicology. Plants 10 (12), 2751. 10.3390/plants10122751 34961221 PMC8703329

[B125] Wang Y. Li J. Li N. (2021). Phytochemistry and pharmacological activity of plants of Genus curculigo: an updated review since 2013. Molecules 26 (11), 3396. 10.3390/molecules26113396 34205154 PMC8199960

[B126] Wang Z. Yang Y. Wang Q. Wang L. Zhao Y. Qian X. (2026). Pathological mechanisms and clinical research progress of endothelial dysfunction. Front. Cardiovasc. Med. 13, 1749548. 10.3389/fcvm.2026.1749548 41717580 PMC12913529

[B127] Wankhade V. Khalekar J. (2012). Effect of MAK-4, an herbal supplement on some biochemical parameters of serum in mice. Asian J. Appl. Sci. Technol. 1 (1), 9–12. 10.51983/ajsat-2012.1.1.681

[B128] Warrier R. R. Priya S. M. Kalaiselvi R. (2021). Gmelina arborea-an indigenous timber species of India with high medicinal value: a review on its pharmacology, pharmacognosy and phytochemistry. J. Ethnopharmacol. 267, 113593. 10.1016/j.jep.2020.113593 33217516

[B129] World Health Organisation (WHO) (2021). Cardiovascular diseases (CVDs). Available online at: https://www.who.int/news-room/fact-sheets/detail/cardiovascular-diseases-(cvds.

[B130] World Health Organisation (WHO) (2022). WHO establishes the global centre for traditional medicine in India. Available online at: https://www.who.int/news/item/25-03-2022-who-establishes-the-global-centre-for-traditional-medicine-in-india.

[B131] Zanella I. DiLorenzo R. DiLorenzo D. (2015). Effects of the dietary supplement MAK-4 on oxidative stress parameters: a “three-cases” report. Open access libr. J. 2:e2150 02, 1–8. 10.4236/oalib.1102150

[B132] Zhu C. Li L. Zhao M. Li J. Gao H. Li H. (2025). Risk of premature cardiovascular disease and all-cause mortality in young adults, association with risk factor prevalence early in life. BMC Cardiovasc. Disord. 25 (1), 352. 10.1186/s12872-025-04814-5 40335893 PMC12057125

