# Supplementary material for: Cardioprotective potential of Maharishi Amrit Kalash: a scoping review of evidence from experimental and clinical studies
Source: Front Pharmacol. 2026 May 14;17:1799117. doi: 10.3389/fphar.2026.1799117 (PMC13216017; doi:10.3389/fphar.2026.1799117)
Supplement: Supplementary file 1 [file Supplementaryfile1.docx]

Cardioprotective Potential of Maharishi Amrit Kalash: A Scoping Review of Evidence from Experimental and Clinical Studies

Radha Singh^1^, Robert Schnieder^2^, Richa Srivastava^1^ Rini Vohra^1,3*^,

1. Maharishi Ayurveda Products Private Limited, Noida, U.P, India-201306
2. College of Integrative Medicine, Maharishi International University, Fairfield, IA
3. Maharishi University of Information Technology, Noida, Uttar Pradesh India

* Corresponding Author: Dr. Rini Vohra

Email id: [dr.rini@naharishiayurveda.global](mailto:dr.rini@naharishiayurveda.global); [rinievohra@gmail.com](mailto:rinievohra@gmail.com)

**Table S1. Scoping Review (PRISMA-ScR) Checklist**

| **SECTION** | **ITEM** | **PRISMA-ScR CHECKLIST ITEM** | **Yes/No** |
| --- | --- | --- | --- |
| **TITLE** | | | |
| Title | 1 | Identify the report as a scoping review. | Yes |
| **ABSTRACT** | | | |
| Structured summary | 2 | Provide a structured summary that includes (as applicable): background, objectives, eligibility criteria, sources of evidence, charting methods, results, and conclusions that relate to the review questions and objectives. | Yes |
| **INTRODUCTION** | | | |
| Rationale | 3 | Describe the rationale for the review in the context of what is already known. Explain why the review questions/objectives lend themselves to a scoping review approach. | Yes |
| Objectives | 4 | Provide an explicit statement of the questions and objectives being addressed with reference to their key elements (e.g., population or participants, concepts, and context) or other relevant key elements used to conceptualize the review questions and/or objectives. | Yes |
| **METHODS** | | | |
| Protocol and registration | 5 | Indicate whether a review protocol exists; state if and where it can be accessed (e.g., a Web address); and if available, provide registration information, including the registration number. | Yes |
| Eligibility criteria | 6 | Specify characteristics of the sources of evidence used as eligibility criteria (e.g., years considered, language, and publication status), and provide a rationale. | Yes |
| Information sources* | 7 | Describe all information sources in the search (e.g., databases with dates of coverage and contact with authors to identify additional sources), as well as the date the most recent search was executed. | Yes |
| Search | 8 | Present the full electronic search strategy for at least 1 database, including any limits used, such that it could be repeated. | Yes |
| Selection of sources of evidence† | 9 | State the process for selecting sources of evidence (i.e., screening and eligibility) included in the scoping review. | Yes |
| Data charting process‡ | 10 | Describe the methods of charting data from the included sources of evidence (e.g., calibrated forms or forms that have been tested by the team before their use, and whether data charting was done independently or in duplicate) and any processes for obtaining and confirming data from investigators. | Yes |
| Data items | 11 | List and define all variables for which data were sought and any assumptions and simplifications made. | Yes |
| Critical appraisal of individual sources of evidence§ | 12 | If done, provide a rationale for conducting a critical appraisal of included sources of evidence; describe the methods used and how this information was used in any data synthesis (if appropriate). | Yes |
| Synthesis of results | 13 | Describe the methods of handling and summarizing the data that were charted. | Yes |
| **RESULTS** | | | |
| Selection of sources of evidence | 14 | Give numbers of sources of evidence screened, assessed for eligibility, and included in the review, with reasons for exclusions at each stage, ideally using a flow diagram. | Yes |
| Characteristics of sources of evidence | 15 | For each source of evidence, present characteristics for which data were charted and provide the citations. | Yes |
| Critical appraisal within sources of evidence | 16 | If done, present data on critical appraisal of included sources of evidence (see item 12). | Yes |
| Results of individual sources of evidence | 17 | For each included source of evidence, present the relevant data that were charted that relate to the review questions and objectives. | Yes |
| Synthesis of results | 18 | Summarize and/or present the charting results as they relate to the review questions and objectives. | Yes |
| **DISCUSSION** | | | |
| Summary of evidence | 19 | Summarize the main results (including an overview of concepts, themes, and types of evidence available), link to the review questions and objectives, and consider the relevance to key groups. | Yes |
| Limitations | 20 | Discuss the limitations of the scoping review process. | Yes |
| Conclusions | 21 | Provide a general interpretation of the results with respect to the review questions and objectives, as well as potential implications and/or next steps. | Yes |
| **FUNDING** | | | |
| Funding | 22 | Describe sources of funding for the included sources of evidence, as well as sources of funding for the scoping review. Describe the role of the funders of the scoping review. | Yes |

**Table S2. Sources used to collect information**

| **Search Engine** | **Terms used** |
| --- | --- |
| PubMed | Maharishi amrit kalash OR amrit kalash OR amrit nectar OR (MAK-4 OR MAK-5]) OR (MAK-7) OR (maharishi, antioxidants, free radicals, CVDs, heart disease, Atherosclerosis, plaque, LDL oxidation. |
| Google Scholar | "Maharishi Amrit Kalash", Ayurveda, Cardiovascular Diseases (CVDs), antioxidants, plaque, atheroma |
|  | "Amrit Kalash", MAK-4, MAK-5, MAK and atherosclerosis |
| AYUSH Research Portal | Maharishi Amrit Kalash, MAK-4, MAK-5 |
| Clinical Trials.gov | Maharishi Amrit Kalash, MAK-4, MAK-5 |
| CTRI | Maharishi Amrit Kalash, MAK-4, MAK-5 |
| DHARA | Maharishi Amrit Kalash, MAK-4, MAK-5 |
| EBSCO | Maharishi Amrit Kalash, Amrit Kalash, MAK-4, MAK-5 |
| Github | Maharishi Amrit Kalash, Amrit Kalash, MAK-4, MAK-5 |
| **Other Sources** | |
| Other Web Resources (http://maharishi-india.org/healthresearch.htm) | - Mashour NH, Lin GI, Frishman WH. Herbal medicine for the treatment of cardiovascular disease: clinical considerations. Arch Intern Med. 1998;158(20):2225-2234. doi:10.1001/archinte.158.20.2225 - Rajalakshmi S, Sharma RK. Effect of Maharishi Amrit Kalash (MAK-4) on mRNAs coding for hepatic glycosyltransferases in the rat. Proc Am Assoc Cancer Res. 1992; 33:13–16. - Panganamala, R. V., & Sharma, H. M. (1991). Antioxidant and Antiplatelet Properties of Maharishi Amrit Kalash (M-4) in Hypercholesterolemic Rabbits. Proceedings of the 9th International Symposium on Atherosclerosis, International Atherosclerosis Society, Rosemont, Illinois, October 6-11, 1991, 188. Abstracts 110 and 111. - Walton, K. G., Olshansky, B., Helene, E., & Schneider, R. H. (2014). Trials of Maharishi Ayurveda for cardiovascular disease: A pooled analysis of outcome studies with carotid intima-media thickness. Journal of preventive cardiology, 4(1), 615–623. - Sharma HM, Triguna BD, Chopra D. Maharishi Ayur-Veda: modern insights into ancient medicine [published correction appears in JAMA 1991 Aug 14;266(6):798]. JAMA. 1991;265(20):2633-2637. |
| Maharishi Ayurveda Research Summary | - Bondy, S. C., Hernandez, T. M., & Mattia, C. (1994).  Antioxidant properties of two ayurvedic herbal preparations. Biochemical Archives, 10(1), 25–31. - Sharma, H.M., Hanna, A.N., Titterington, L.C., Lubow, G.P., Ralph, E.S.The antioxidant activity of Maharishi Amrit Kalash (MAK-4 and MAK-5), estrogen and Vitamin C. Presented at Scientific Conference on Atherosclerosis, Thrombosis, and Proliferation, American Heart Association, Orlando, FL, February 23-26, 1994. |
